# Glutamine deficiency induces DNA alkylation damage and sensitizes cancer cells to alkylating agents through inhibition of ALKBH enzymes

**DOI:** 10.1371/journal.pbio.2002810

**Published:** 2017-11-06

**Authors:** Thai Q. Tran, Mari B. Ishak Gabra, Xazmin H. Lowman, Ying Yang, Michael A. Reid, Min Pan, Timothy R. O’Connor, Mei Kong

**Affiliations:** Department of Cancer Biology, Beckman Research Institute of the City of Hope, Duarte, California, United States of America; University of California at Los Angeles, United States of America

## Abstract

Driven by oncogenic signaling, glutamine addiction exhibited by cancer cells often leads to severe glutamine depletion in solid tumors. Despite this nutritional environment that tumor cells often experience, the effect of glutamine deficiency on cellular responses to DNA damage and chemotherapeutic treatment remains unclear. Here, we show that glutamine deficiency, through the reduction of alpha-ketoglutarate, inhibits the AlkB homolog (ALKBH) enzymes activity and induces DNA alkylation damage. As a result, glutamine deprivation or glutaminase inhibitor treatment triggers DNA damage accumulation independent of cell death. In addition, low glutamine-induced DNA damage is abolished in ALKBH deficient cells. Importantly, we show that glutaminase inhibitors, 6-Diazo-5-oxo-L-norleucine (DON) or CB-839, hypersensitize cancer cells to alkylating agents both in vitro and in vivo. Together, the crosstalk between glutamine metabolism and the DNA repair pathway identified in this study highlights a potential role of metabolic stress in genomic instability and therapeutic response in cancer.

## Author summary

Cancer cells residing within the intratumoral microenvironment are subject to severe glutamine shortages. Herein, we provide mechanistic insight by which glutamine deficiency leads to cellular sensitivity to alkylating agents. We find that glutamine deficiency inhibits the DNA repair activity of the ALKBH enzymes, leading to accumulation of DNA alkylation damage and thereby increasing cellular sensitivity to alkylating agents. This study provides a critical molecular basis to combine glutaminase inhibitors with alkylating agents for more effective treatment of cancers. These findings extend our understanding of the role of metabolic stress, in particular glutamine deficiency, in tumor development and therapeutic response.

## Introduction

Metabolic alterations exhibited by cancer cells can potentiate tumorigenesis and promote cell survival [1,2]. Unlike normal cells, cancer cells favor aerobic glycolysis, also known as the Warburg effect, to support rapid proliferation [3]. As most glucose is converted into lactate, cancer cells become heavily dependent on glutamine as a major carbon and nitrogen source [4]. Glutamine metabolism supports rapidly proliferating cells by facilitating the biosynthesis of different amino acids and nucleotides [3,5]. Moreover, glutamine supports the increased energetic demand and suppresses accumulated reactive oxygen species (ROS) exhibited in cancer cells [6]. Specifically, glutamine is diverted to synthesize the tricarboxylic acid (TCA) cycle intermediate, alpha-ketoglutarate (αKG), to replenish the truncated TCA cycle and maintain healthy NADH and NADPH levels [6–8]. Moreover, the amino acid drives the production of glutathione (GSH), a major antioxidant, to protect cancer cells from ROS accumulation [9]. Inhibition of glutamine metabolism with small molecule inhibitors results in an energetic crisis leading to cellular death in some cancers [10,11].

On the other hand, the increased glutamine uptake in cancer cells coupled with poor vascularization in tumors often leads to severe glutamine shortage in the tumor microenvironment [12,13]. For example, metabolomics studies on human pancreatic cancer patient samples have clearly demonstrated that glutamine, besides glucose, is one of the most depleted metabolites in tumors compared to adjacent healthy tissues [13]. In addition, core regions of solid tumors display extreme glutamine deficiency compared to peripheral regions in melanoma xenografts and transgenic mouse tumors [14]. Interestingly, many cancer cells appear to adapt to this strong metabolic stress through multiple mechanisms, including p53 and IKKβ activation [15–17]. However, it remains unclear how glutamine deficiency observed in tumors impacts tumor development and therapeutic response.

Genomic instability plays a significant role in tumorigenesis and aging [18]. While cellular DNA is constantly exposed to both endogenous and exogenous DNA damaging agents, the damages are regularly repaired by the robust DNA damage repair pathways [19]. The AlkB homolog (ALKBH) enzymes are dioxygenases that directly reverse DNA alkylation damage caused by both endogenous and exogenous sources and help maintain genomic integrity [20,21]. Interestingly, ALKBH overexpression in cancer promotes drug resistance, leading to poor prognosis in multiple cancers [22,23]. For example, ALKBH2 overexpression induces cellular resistance to alkylating agent treatment in glioblastoma and promotes cancer progression in bladder cancer [23,24]. Moreover, ALKBH3 overexpression promotes alkylation damage resistance in prostate cancer and apoptotic resistance in pancreatic cancer [25–27]. In response to DNA alkylation damage, the Fe(II)dependent ALKBH enzymes use αKG as a key substrate to directly remove alkyl groups from DNA adducts [21]. The requirement of αKG by the ALKBH enzymes to repair DNA alkylation damage underlines the potential crosstalk between cellular metabolism and the DNA damage repair pathway. Because glutamine catabolism directly contributes to cellular αKG pools in many cancers [14], it will be of interest to examine whether glutamine deficiency affects the DNA repair function of the αKG-dependent ALKBH enzymes.

In this study, we found that glutamine deficiency inhibits the ALKBH enzymes from repairing DNA alkylation damage, leading to DNA damage in the absence of the genotoxic agent. Importantly, our results demonstrate that targeting glutamine metabolism significantly sensitizes cancer cells to alkylating agent treatments both in vitro and in vivo. Together, our study reveals a previously unidentified role of glutamine deficiency in modulating the DNA damage response and provides a molecular basis for combinational therapy using glutaminase inhibitors and alkylating agents.

## Results

### Glutamine deficiency specifically triggers DNA damage accumulation independent of cell death

**Fig 1:**
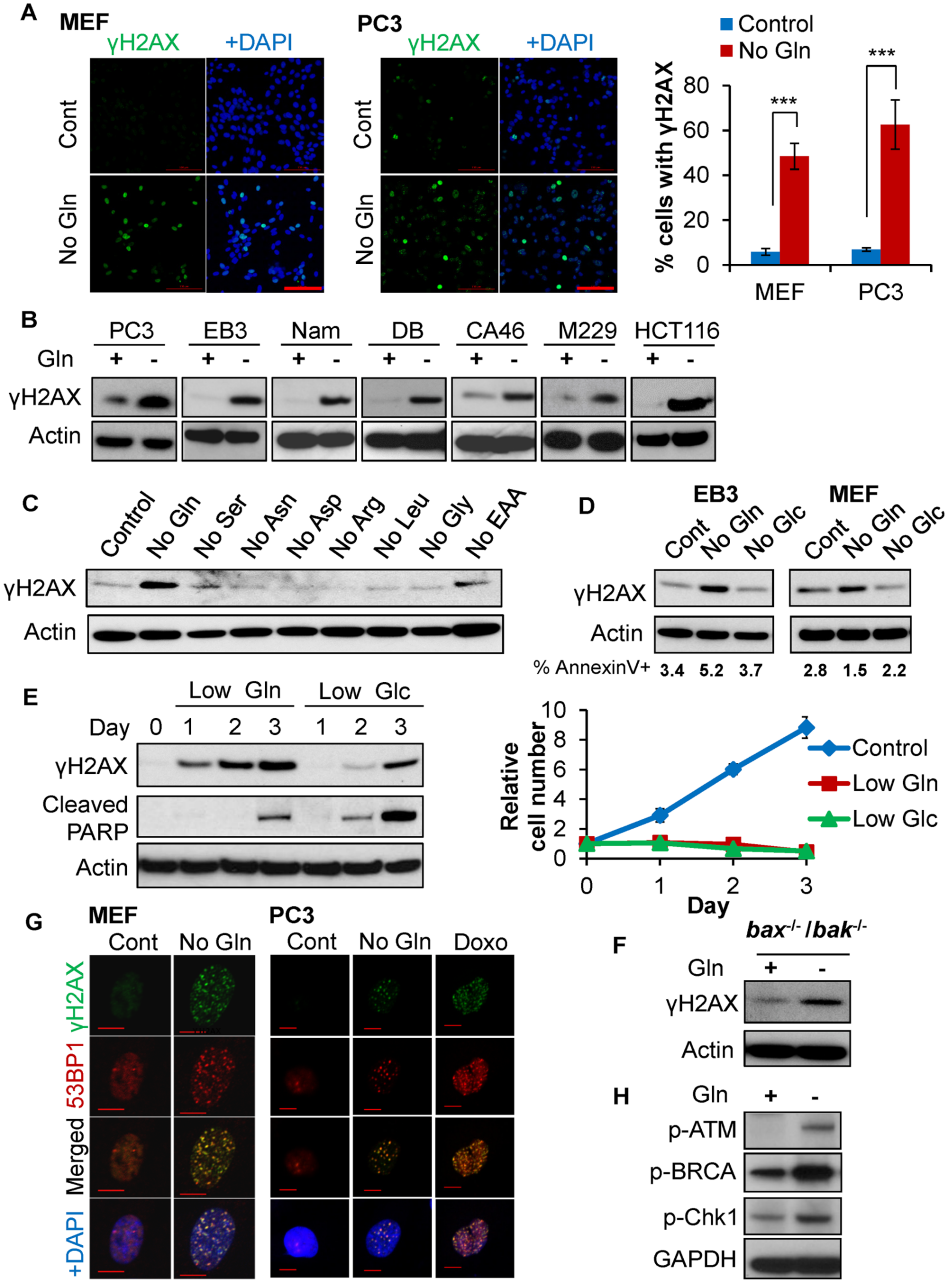
Glutamine deficiency specifically triggers DNA damage accumulation independent of cell death. (A) MEF and PC3 cells were cultured in complete (Cont) or glutamine free medium (No Gln) for 24 hours. Cells were fixed for immunofluorescence with γH2AX antibody and DAPI. Scale bar 100 μm. Data represent mean ± SD of 4 independent cell cultures, *** *P* < 0.001, shown is the percentage of cells showing >10 foci. (B) The indicated cancer cell lines were cultured in complete or glutamine-free medium. (C) MEF cells were cultured in medium deprived of the indicated amino acid for 48 hours. (D) EB3 and MEF cells were cultured in complete medium, glutamine-free or glucose-free medium for 6 hours. Early apoptotic cell death was determined by AnnexinV staining with flow cytometry. (E) MEF cells were cultured in either low glutamine (0.1 mM) or low glucose (1 mM) for the indicated time points; relative cell growth was determined by CellTiter-Glo assay. (B-E) The treated cells were lysed for immunoblotting using indicated antibodies. (F) *Bak*^-/-^ /*Bax* ^-/-^ MEF cells were starved of glutamine for 48 hours, and cells were lysed for immunoblotting. (G) MEF and PC3 cells were cultured in complete or glutamine-free medium overnight. PC3 cells were also treated with 3.4 μM Doxo for 4 hours to induce DNA DSB. The cells were fixed for immunofluorescence using the indicated antibodies. Scale bar 10 μm. (H) EB3 cells were cultured in complete or glutamine-free medium overnight; cells were lysed for western blot analysis using the indicated antibodies. Arg, arginine; Asn, asparagine; Asp, aspartate; Cont, control; Doxo, doxorubicin; DSB, double strand breaks; EAA, essential amino acids; GAPDH, Glyceraldehyde 3-phosphate dehydrogenase; Glc, glucose; Gln, glutamine; Gly, glycine; Leu, leucine; MEF, mouse embryonic fibroblast; Nam, Namalwa; p-ATM, ataxia-telangiectasia mutated; p-Chk1, checkpoint kinase 1; Ser, serine.

To determine the impact of glutamine deficiency on genomic integrity, we first asked whether glutamine depletion leads to accumulation of DNA damage. Mouse embryonic fibroblast (MEF) cells and prostate cancer PC3 cells were cultured in complete or glutamine free medium for 24 hours followed by immunofluorescence for γH2AX, an established biomarker for DNA damage [28]. We found that glutamine withdrawal led to a significant induction of γH2AX in both MEF and PC3 cells (Fig 1A). Using immunoblotting, we found that low glutamine-induced γH2AX signal was both dose dependent and time dependent (S1A and S1B Fig). Consistently, glutamine withdrawal resulted in striking accumulation of DNA damage as marked by γH2AX in multiple cancer cell lines (Fig 1B). Compared to glutamine deprivation, starvation of any other amino acid failed to induce γH2AX (Fig 1C). Unlike glutamine deprivation, deprivation of glucose for the same amount of time failed to induce γH2AX in both MEF and EB3 cells, supporting that glutamine deficiency specifically induces DNA damage (Fig 1D).

Although prolonged glutamine starvation triggers cell death [29], γH2AX induction was detected when the majority of cells were still viable (Fig 1D). To further confirm that glutamine deficiency induces γH2AX independent of cell cycle arrest and cell death, we assessed γH2AX levels in MEF cells cultured in low glutamine conditions or low glucose conditions over time. Glutamine deficiency dramatically induced γH2AX on day 1 and peaked on day 2 when apoptotic cell death was not detected. In contrast, the γH2AX induction in low glucose conditions was marginal compared to low glutamine conditions and possibly caused by the activation of apoptotic cell death as marked by the induced cleaved poly (adenosine diphosphate-ribose) polymerase (PARP; Fig 1E). Interestingly, we found that low glutamine and low glucose inhibited cell proliferation to a similar extent, suggesting that cell cycle arrest may not significantly contribute to the DNA damage (Fig 1E). In addition, immunofluorescence for γH2AX and the apoptotic marker, cleaved caspase-3, further revealed that the majority of γH2AX-positive cells were not apoptotic cells (S1C Fig). To further confirm that glutamine deprivation-induced γH2AX is independent of apoptosis, we used *Bax*^-/-^ /*Bak*^-/-^ MEF cells, which lack apoptotic machinery (S1D Fig) [30]. We found that glutamine deprivation still promotes γH2AX in apoptosis-deficient cells (Fig 1F). Lastly, although γH2AX is a reliable marker for DNA damage, γH2AX is also induced during DNA replication stress [31] and cellular death [32]. To examine whether glutamine depletion triggers physical breaks in DNA, we performed immunofluorescence to detect γH2AX and 53BP1 colocalizations, which occur at DNA double strand breaks (DSB) [33,34]. Similar to doxorubicin (Doxo) treatment that induced DSBs, glutamine depletion triggered γH2AX and 53BP1 foci colocalization in both MEF and PC3 cells, demonstrating that glutamine depletion can result in DSBs (Fig 1G). Consistently, DNA damage–induced signaling pathways are increased upon glutamine deprivation (Fig 1H). Taken together, these results demonstrate that glutamine deficiency specifically triggers DNA damage accumulation independent of cell death.

### Inhibition of glutamine metabolism with a glutaminase inhibitor triggers DNA damage accumulation both in vitro and in vivo

**Fig 2:**
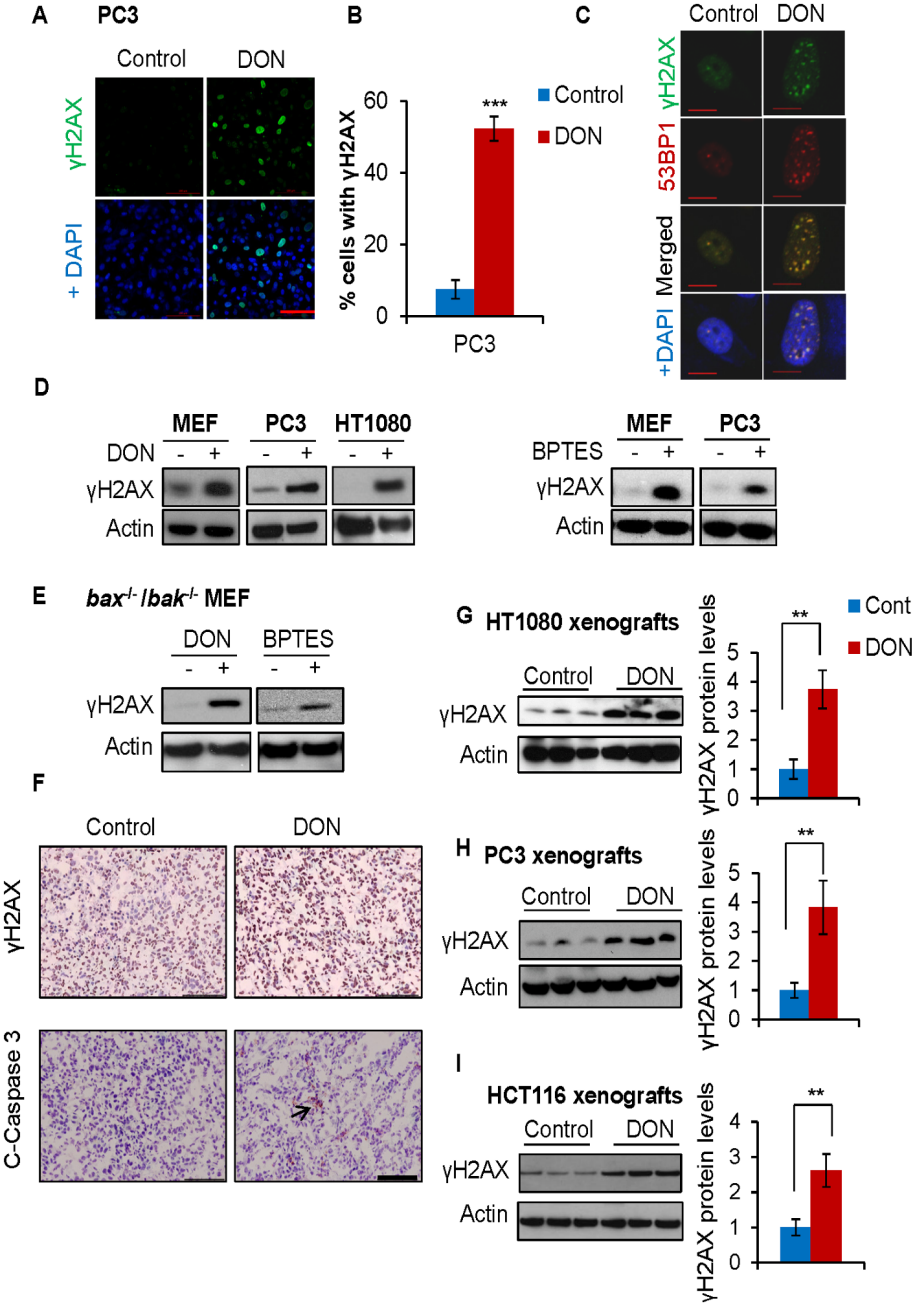
Inhibition of glutamine metabolism with a glutaminase inhibitor triggers DNA damage accumulation both in vitro and in vivo. (A-B) PC3 cells were treated with 50 μM DON for 48 hours. The cells were fixed for immunofluorescence using γH2AX antibody. Data represent mean ± SD of 4 independent cell cultures, *** *P* < 0.001, shown is the percentage of cells showing >10 foci. Scale bar 100 μm. (C) PC3 cells were treated with 50 μM DON for 48 hours, and the cells were fixed for immunofluorescence using the indicated antibodies. Scale bar 10 μm. (D) MEF, PC3, or HT1080 cells were treated with 50 μM DON or 50 μM BPTES for 48 hours. (E) *Bak*^-/-^ /*Bax* ^-/-^ MEF cells were treated with 50 μM DON or 50 μM BPTES for 48 hours, and the cells were lysed for immunoblotting using the indicated antibodies. (F-G) Mice bearing HT1080 xenograft tumors were treated with 10 mg/kg DON every other day for 2 weeks. The tumors were harvested and fixed for IHC and immunoblotting using the indicated antibodies. (H-I) Mice bearing PC3 xenograft tumors were treated with 5 mg/kg DON and mice bearing HCT116 p53-/- xenograft tumors were treated with 15 mg/kg DON every other day for 2 weeks. The control- and DON-treated tumors were harvested and lysed for immunoblotting using the indicated antibodies. Each lane represents an individual tumor, and immunoblots were quantified by ImageJ and normalized to the control group. Data represent mean ± SD of 3 individual tumors, ** *P* < 0.01. DON, 6-Diazo-5-oxo-L-norleucine; IHC, immunohistochemistry; MEF, mouse embryonic fibroblast.

Glutamine withdrawal not only hinders glutaminolysis but also affects other functions of intracellular glutamine including, but not limited to, protein synthesis and mTOR regulation [35,36]. To determine whether inhibition of glutaminolysis alone can trigger DNA damage, we assayed for the induction of γH2AX by immunofluorescence in PC3 cells after treatment with the glutaminase inhibitor, 6-Diazo-5-oxo-L-norleucine (DON). The drug treatment significantly induces γH2AX in PC3 cells (Fig 2A and 2B). In addition, the glutaminase inhibition in PC3 cells induced robust DSBs as indicated by the γH2AX and 53BP1 colocalization (Fig 2C). Moreover, different glutaminase inhibitors, such as DON and Bis-2-(5-phenylacetamido-1,3,4-thiadiazol-2-yl)ethyl sulfide (BPTES), induced γH2AX in multiple cell lines (Fig 2D). Importantly, DON and BPTES also induced DNA damage in apoptosis-deficient cells (Fig 2E). To determine whether inhibition of glutamine metabolism also triggers DNA damage in vivo, we treated HT1080 xenograft tumors with DON. Immunohistochemistry (IHC) staining revealed that DON treatment dramatically induced γH2AX levels, but not cleaved caspase-3 levels (Fig 2F). Using immunoblotting, we also confirmed dramatic γH2AX induction in the DON-treated tumors compared to control-treated tumors in HT1080, PC3, and HCT116 xenograft tumors (Fig 2G–2I). Therefore, similar to glutamine starvation, inhibition of glutamine metabolism also triggered DNA damage both in vitro and in vivo.

### Glutamine deficiency–induced DNA damage is αKG dependent

**Fig 3:**
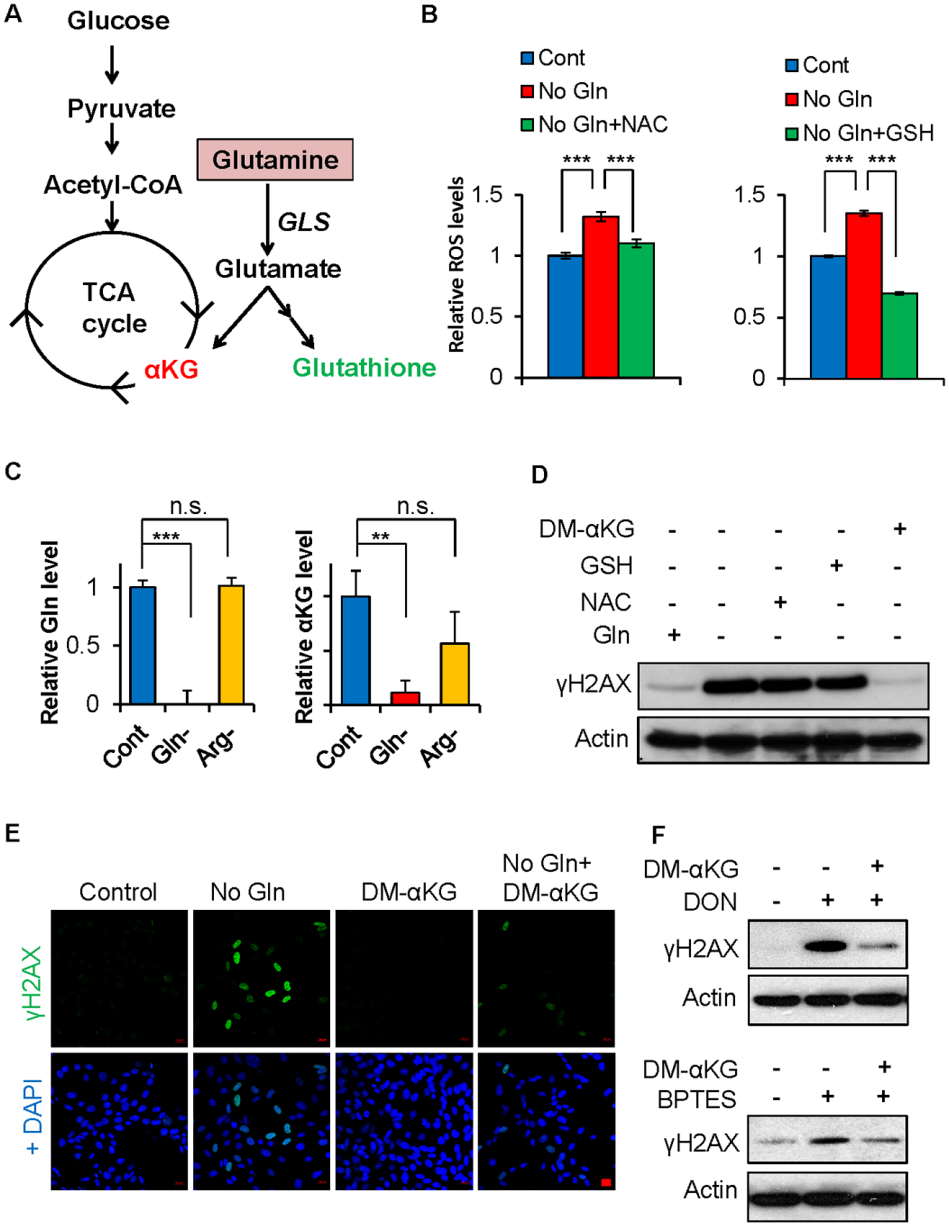
Glutamine deficiency–induced DNA damage is αKG dependent. (A) Diagram of glutamine metabolism in cancer. (B) EB3 cells were cultured in complete, glutamine-free medium or glutamine-free medium supplemented with 10 mM NAC or 15 mM GSH for 6 hours. The cells were harvested for flow cytometry analysis with dihydroethidium dye to determine relative ROS levels normalized to the control; data represent mean ± SD of minimum 3 independent cell cultures, *** *P* < 0.001. (C) MEF cells were cultured in glutamine-free medium or arginine-free medium (negative control) for 6 hours. Metabolites were extracted for mass spectrometry analysis to measure the indicated total intracellular metabolites, normalized to the control. Data represent mean ± SD of 3 independent cell cultures, ***P < 0.001. (D) EB3 cells were cultured in complete or glutamine-free medium supplemented with 10 mM NAC, 15 mM GSH, or 7 mM DM-αKG for 6 hours. Cells were lysed for immunoblotting with the indicated antibodies. (E) MEF cells were cultured in complete or glutamine-free medium supplemented with 3.5 mM DM-αKG overnight, the cells were fixed for immunofluorescence with the indicated antibodies. Scale bar 20 μm. (F) EB3 cells were treated with 50 μM DON or 50 μM BPTES in control medium or medium supplemented with 3.5 mM DM-αKG for 48 hours, cells were lysed for immunoblotting with the indicated antibodies. αKG, alpha-ketoglutarate; Arg, arginine; DM-αKG; dimethyl-αKG; DON, 6-Diazo-5-oxo-L-norleucine; Gln, glutamine; GLS, glutaminase; GSH, glutathione; MEF, mouse embryonic fibroblast; NAC, N-acetyl cysteine; n.s., not significant; TCA, tricarboxylic acid.

Cancer cells convert glutamine into glutamate that can be subsequently used to synthesize the antioxidant GSH and αKG (Fig 3A) [7,9]. To test whether glutamine deficiency induces ROS that contributes to DNA damage, we measured ROS levels after glutamine withdrawal and found that glutamine starvation rapidly promoted ROS accumulation, which was fully abolished with an antioxidant supplement of either N-acetyl cysteine (NAC) or GSH (Fig 3B). Next, we confirmed that glutamine depletion and glutaminase inhibitor treatment diminished intracellular αKG levels in cells, consistent with previous studies (Fig 3C and S2 Fig) [7,37]. However, despite the ability of both NAC and GSH to block ROS induction, they failed to inhibit the DNA damage caused by glutamine deficiency (Fig 3D). Interestingly, supplementing with dimethyl-αKG (DM-αKG) completely abolished low glutamine-induced DNA damage, suggesting that αKG plays a critical role in maintaining genomic integrity in glutamine-depleted cells (Fig 3D and 3E). Consistently, DM-αKG reversed the γH2AX induction upon glutaminase inhibitor treatment (Fig 3F). Thus, the induction of DNA damage upon glutamine deprivation or glutaminase inhibitor treatment is mediated by the depletion of intracellular αKG.

### Glutamine deficiency inhibits ALKBH activity and induces endogenous DNA alkylation damage

**Fig 4:**
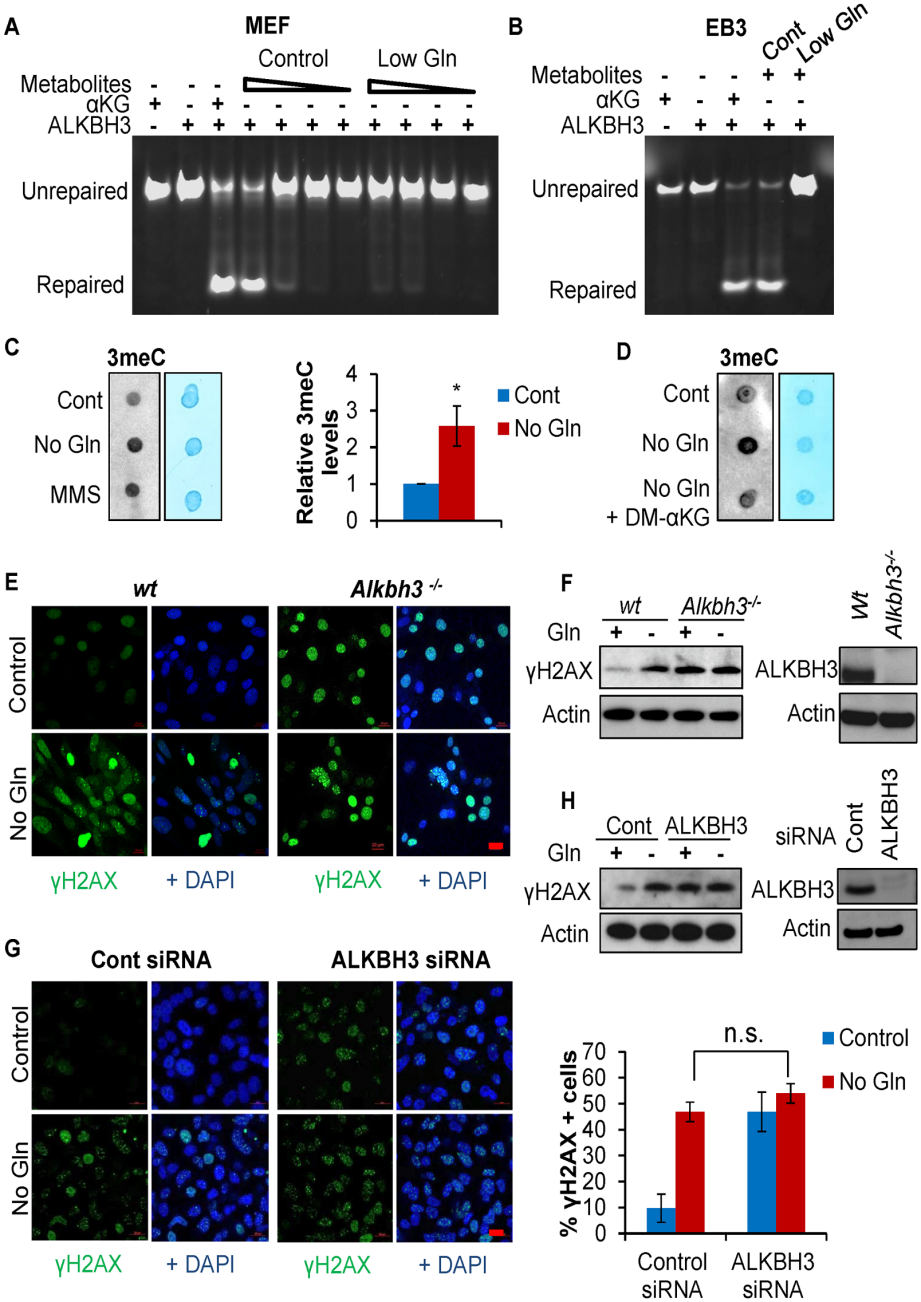
Glutamine deficiency inhibits ALKBH activity and induces endogenous DNA alkylation damage. (A) MEF cells were cultured in complete or 0.1 mM glutamine medium for 48 hours, and methanol was used to extract intracellular metabolites from an equal number of viable cells. To perform the ALKBH3 enzymatic activity assay, the extracted metabolites or αKG (positive control) were incubated with the 3meC-containing DNA probe and recombinant ALKBH3. (B) EB3 cells were cultured in complete or low-glutamine medium for 24 hours, and the cells were then processed as in Fig 4A. (C) MEF cells were cultured in complete, glutamine-free medium for 48 hours or treated with 2 mM MMS for 1 hour. Genomic DNA was extracted to perform dot blot analysis using the 3meC antibody. DNA loading was assessed by methylene blue dye. Data represent mean ± SD of 4 independent cell cultures, * *P* < 0.05. (D) Cells were cultured in complete, glutamine-free medium or glutamine-free medium supplemented with 3.5 mM DM-αKG for 48 hours followed by dot blot analysis. (E-F) Wild-type MEF or *Alkbh3*^-/-^ MEF cells were cultured in complete or glutamine-free medium overnight; cells were fixed for immunofluorescence and immunoblotting by using the indicated antibodies. (G-H) PC3 cells were transfected with ALKBH3 siRNA twice. Four days after transfection, control PC3 cells and ALKBH3 knockdown cells were cultured in complete or glutamine free medium for 2 days; cells were fixed for immunofluorescence and immunoblotting by using the indicated antibodies. Scale bar 20 μm. Data represent mean ± SD from 2 independent cell cultures; shown is the percentage of cells showing >10 foci. 3meC, 3-methyl cytosine; αKG, alpha-ketoglutarate; ALKBH, AlkB homolog; Cont, control; DM- αKG, dimethyl- αKG; Gln, glutamine; MEF, mouse embryonic fibroblast; MMS, methyl methanesulfonate; n.s., not significant; siRNA, small interfering RNA; wt, wild-type.

ALKBH enzymes require αKG as an essential cofactor to repair DNA alkylation damage and maintain genomic integrity [38]. Because glutamine deficiency leads to dramatic reduction of intracellular αKG levels, we next investigated if such metabolic stress inhibits the DNA damage repair by ALKBH enzymes. We first performed enzymatic activity assays for ALKBH3 using extracted intracellular metabolites from cells cultured in complete medium or low-glutamine medium. Both αKG and the extracted metabolites from control cells robustly promoted the ALKBH3 enzyme to remove methyl groups from 3-methyl cytosine (3meC) adducts (Fig 4A and 4B). In contrast, extracted metabolites from glutamine-deprived cells failed to support ALKBH3 enzymatic activity in both MEF and EB3 cell lines, suggesting that glutamine deprivation inhibits the activity of ALKBH enzymes through the alteration of intracellular metabolites, but not other signaling pathways. To further determine whether glutamine deficiency inhibits ALKBH enzymes to repair alkylated DNA adducts in the cells, we performed dot blot analysis with a 3meC-specific antibody to measure global genomic 3meC in cells cultured in complete medium or glutamine-free medium. As shown in Fig 4C, glutamine deficiency significantly induced 3meC in cells to a similar extent as methyl methanesulfonate (MMS) treatment, consistent with the ALKBH activity and γH2AX levels. Importantly, glutamine deficiency–induced 3meC accumulation was largely rescued by DM-αKG supplementation (Fig 4D). Furthermore, glutamine deprivation failed to induce further alkylation damage, as marked by 3meC levels in the ALKBH3 depleted cells (S3A Fig).

To test the hypothesis that low glutamine-induced DNA damage is caused by ALKBH inhibition, we cultured *Alkbh2*^*-/-*^ MEF, *Alkbh3*^*-/-*^ MEF, and paired wild-type MEF cells in glutamine-free medium or complete medium overnight. We found that *Alkbh2*^*-/-*^ and *Alkbh3*^*-/-*^ MEF cells exhibited higher γH2AX levels in complete medium compared to the wild-type MEF cells (Fig 4E and 4F and S3B Fig), suggesting that ALKBH actively protects cells from endogenous alkylating agents. Importantly, glutamine depletion failed to further promote γH2AX induction in ALKBH3 knockout or ALKBH2 knockout MEF cells compared to the wild-type cells (Fig 4E and 4F and S3B Fig). Similarly, we found no further increase in γH2AX upon glutamine starvation in ALKBH3 knockdown PC3 cancer cells (Fig 4G and 4H). In contrast, treatment of the topoisomerase inhibitor camptothecin (CPT) further promoted DNA damage in the ALKBH3 knockdown cells (S3C Fig). In addition, we found that DM-αKG, which rescued low glutamine-induced DNA damage in control cells, failed to rescue the DNA damage in ALKBH-deficient cells (S4A and S4B Fig). Together, these data indicate that glutamine depletion, by reducing the key cofactor αKG, inhibits the ALKBH enzyme from repairing endogenous DNA alkylation damage.

### Glutamine deficiency sensitizes cells to alkylating-agent–induced DNA damage

**Fig 5:**
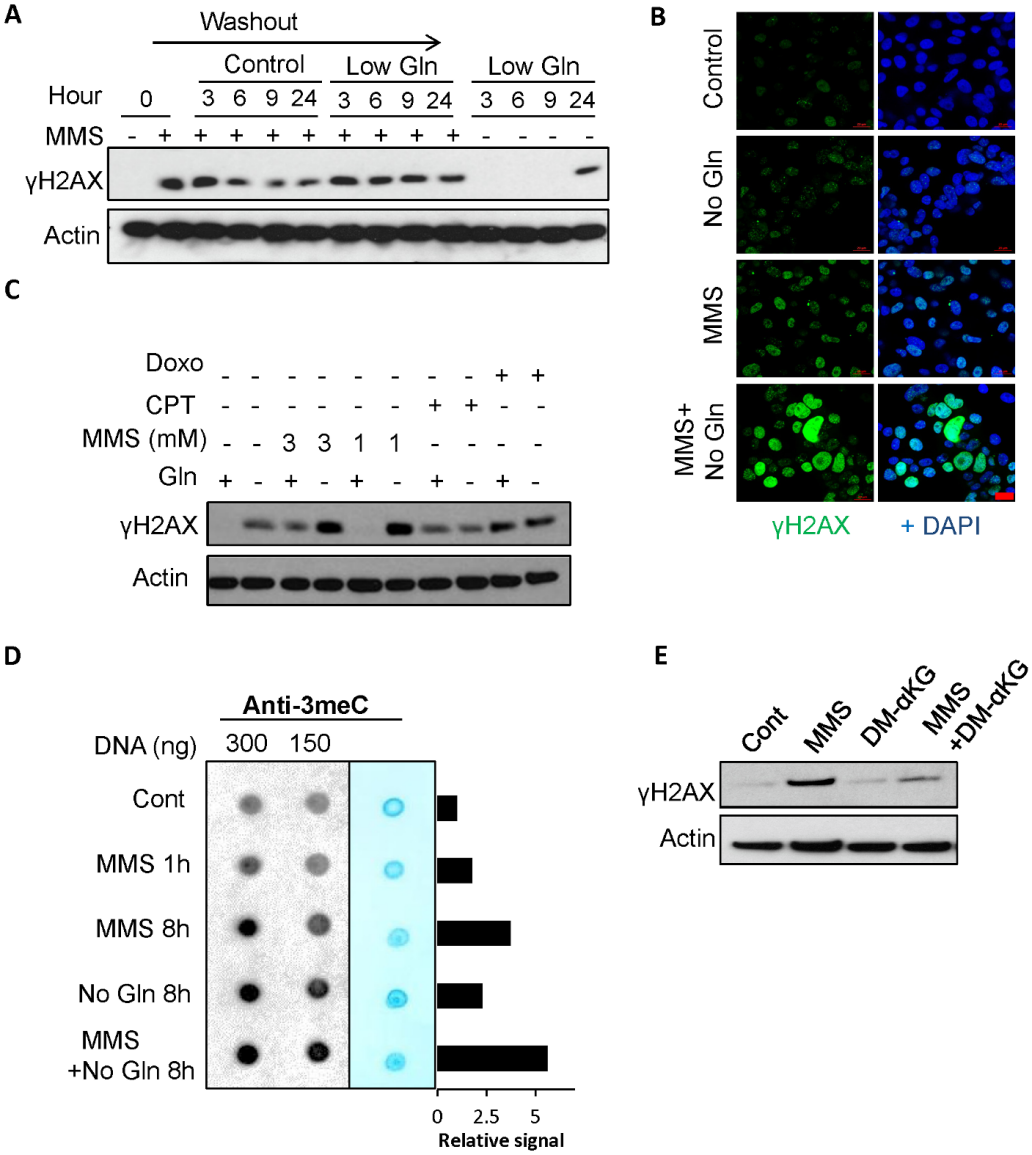
Glutamine deficiency sensitizes cells to alkylating-agent–induced DNA damage. (A) MEF cells were treated with 1 mM MMS for 1 hour, washed, and subsequently cultured in complete medium with 2 mM Gln or low glutamine medium with 0.1 mM Gln for the indicated time points. Cells were lysed for immunoblotting with the indicated antibodies. (B) PC3 cells were treated with 1 mM MMS for 1 hour, washed, and subsequently cultured in complete medium or glutamine-free medium for 48 hours. Cells were fixed for immunofluorescence with the γH2AX antibody and DAPI. Scale bar 20 μm (C) MEF cells were treated with 1 or 3 mM MMS, 5 μM CPT, or 3.4 μM Doxo in complete or low-glutamine medium for 24 hours; cells were lysed for immunoblotting. (D) MEF cells were treated with 1 mM MMS for 1 hour, washed, and subsequently cultured in complete or glutamine-free medium for 8 hours. Genomic DNA was extracted to perform dot blot analysis using the 3meC specific antibody. DNA loading was assessed by methylene blue dye. 3meC signals relative to the control were quantified by ImageJ and normalized to methylene blue. (E) MEF cells were treated with 1 mM MMS for 1 hour, washed, and subsequently cultured in complete medium or medium supplemented with 1 mM DM-αKG for 24 hours. Cells were lysed for immunoblotting using the indicated antibodies. 3meC, 3-methyl cytosine; CPT, camptothecin; DM-αKG, dimethyl-alpha-ketoglutarate; Doxo, doxorubicin; Gln, glutamine; MEF, mouse embryonic fibroblast; MMS, methyl methanesulfonate.

We next sought to determine whether glutamine deficiency can sensitize cells to alkylating-agent–induced DNA damage. We cultured cells that were initially exposed to the alkylating agent, MMS, for 1 hour in complete medium or low glutamine medium. Induction of γH2AX was absent in the nontreated cells but was present after MMS treatment (Fig 5A). For cells cultured in complete medium, the γH2AX signal peaked 3 hours after MMS treatment but diminished after 6 hours upon removal of the drug. In contrast, γH2AX signals in low glutamine conditions remained high after the drug removal even after 9 hours later, while low glutamine alone at 9 hours had no effect on γH2AX (Fig 5A). These results indicate that low glutamine conditions inhibit the ability of ALKBH enzymes to repair DNA damage caused by the alkylating agent. Consistently, immunofluorescence of γH2AX revealed that glutamine deficiency potentiated DNA damage caused by the alkylating agent (Fig 5B). Interestingly, glutamine starvation did not affect the DNA damage caused by other classes of chemotherapy, including the topoisomerase inhibitor Doxo and CPT, suggesting that glutamine deficiency only affects alkylation damage repair, but not other DNA damage repair pathways (Fig 5C). To further determine whether glutamine deficiency attenuates ALKBH activities to repair methylated DNA adducts upon alkylating agent treatment, we cultured alkylating agent–treated cells in either complete or glutamine-free medium and measured global genomic 3meC levels. The 3meC levels increased after MMS treatment for 1 hour. The drug was then washed out and cells were cultured in complete medium or glutamine-free medium for 8 hours. Intriguingly, MMS treatment in combination with glutamine starvation at 8 hours resulted in the highest induction of 3meC compared to the single treatments, highlighting that glutamine deficiency potentiates alkylating-agent–induced DNA damage by directly interfering with the ALKBH enzymes to repair alkylation damage (Fig 5D). Furthermore, the addition of cell permeable DM-αKG largely blocked MMS-induced γH2AX, suggesting that glutamine deficiency potentiates the MMS effect via αKG reduction (Fig 5E).

### Inhibition of glutamine metabolism hypersensitizes cells to alkylating agents

**Fig 6:**
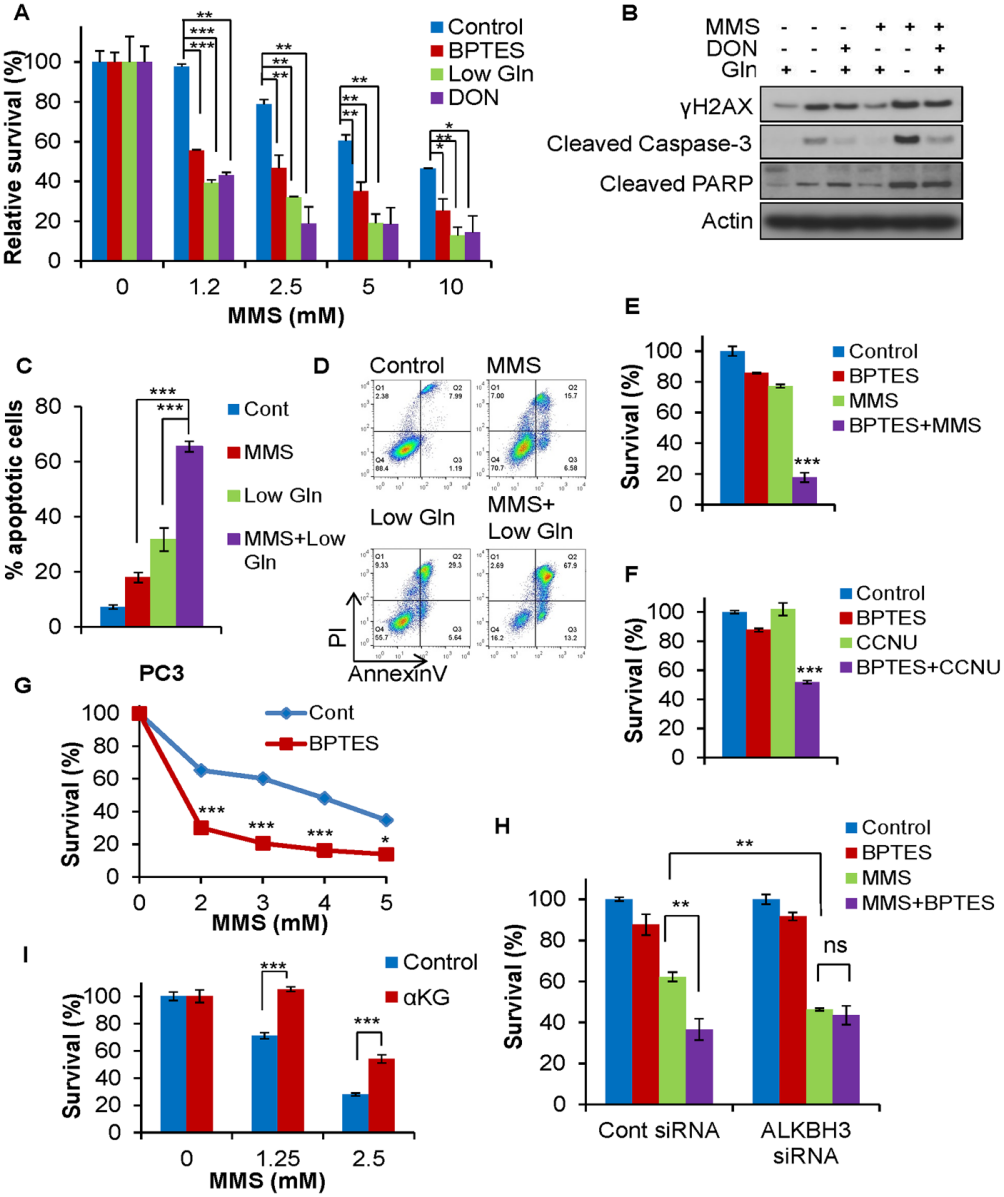
Inhibition of glutamine metabolism hypersensitizes cells to alkylating agents. (A) Ras-transformed MEF cells were treated with the indicated concentration of MMS for 1 hour, washed, and subsequently cultured in 0.1 mM glutamine medium, complete medium supplemented with 20μM BPTES, or 25μM DON for 48 hours. Relative cell survival was assessed by MTS assay and normalized to the control of each group. Data represent mean ± SD of minimum 2 independent cell cultures, * *P* < 0.05, ** *P* < 0.01, *** *P* < 0.001. (B) Cells were treated as in (A) and lysed for immunoblotting with the indicated antibodies. (C-D) Ras-transformed MEF cells were treated with 1 mM MMS for 1 hour and subsequently cultured in complete medium or 0.1 mM glutamine medium for 48 hours. Apoptotic cell death was assessed by flow cytometry with AnnexinV/PI staining. Data represent mean ± SD of 4 independent cell cultures, *** *P* < 0.001. (E) HT1080 cells were treated with 2.5 mM MMS for 1 hour and subsequently cultured in control medium or medium supplemented with 20 μM BPTES for 48 hours. (F) HT1080 cells were treated with 10 μM BPTES, 35μM CCNU, or a combination of BPTES and CCNU. (G) PC3 cells were treated with indicated concentration of MMS for 1 hour, washed, and subsequently cultured in medium supplemented with 25 μM BPTES for 48 hours. (H) Control cells and siRNA-mediated ALKBH3 knockdown cells were treated as in (G) with 2 mM MMS and 25 μM BPTES. (E-H) Relative cell survival was assessed by MTS assay and normalized to the control. Data represent mean ± SD of 3 independent cell cultures, ** *P* < 0.01, *** *P* < 0.001. (I) MEF cells were treated with the indicated concentration of MMS, washed, and subsequently cultured in complete medium or medium supplemented with 1 mM DM-αKG for 24 hours; relative cell survival was assessed by MTS assay, normalized to the control. Data represent mean ± SD of 4 independent cell cultures, *** *P* < 0.001. αKG, alpha-ketoglutarate; CCNU, lomustine; DM-αKG, dimethyl-αKG; DON, 6-Diazo-5-oxo-L-norleucine; Gln, glutamine; MEF, mouse embryonic fibroblast; MMS, methyl methanesulfonate; PARP, poly (adenosine diphosphate-ribose) polymerase; siRNA, small interfering RNA.

Alkylating agents, one of the earliest classes of chemotherapy, remain as frontline therapies for multiple types of aggressive cancer [39]. To investigate the effect of glutamine deficiency on cellular response to alkylating agents, we treated Ras-transformed MEF cells with different glutaminase inhibitors in combination with alkylating agents. Inhibition of glutamine metabolism with either DON or BPTES hyper-sensitized cancer cells to MMS treatment at multiple doses (Fig 6A). Importantly, low glutamine conditions and glutaminase inhibitor treatment had similar effects on cellular response to alkylating agents, suggesting that these glutaminase inhibitors function via inhibition of glutamine catabolism (Fig 6A). Consistently, impairing glutamine metabolism in combination with the alkylating agent resulted in robust apoptotic cell death compared to the single treatment as measured by cleaved caspase-3, cleaved PARP (Fig 6B), and Annexin/PI staining (Fig 6C and 6D). Interestingly, the glutaminase inhibitor treatment failed to sensitize cancer cells to other DNA damaging agents, such as Doxo or CPT, indicating that inhibition of glutamine metabolism only affects methylation damage repairs, but not DSB repairs (S5 Fig). Additionally, we confirmed the striking synergistic killing seen in human fibrosarcoma cells HT1080. The glutaminase inhibitor BPTES significantly sensitized cancer cells to both MMS and the clinical alkylating agent lomustine (CCNU), confirming that the combination works on a broad range of alkylating agents and across a broad range of cancers (Fig 6E and 6F). Overexpression of ALKBH enzyme in prostate cancer and glioblastoma results in cell resistance to alkylating agents, leading to poor prognosis, as shown in multiple studies [22, 24, 27]. Therefore, we further tested whether inhibition of glutamine metabolism can indirectly inhibit ALKBH and restore cancer cell sensitivity to alkylating agents. Significantly, the combination treatment of MMS and BPTES resulted in a striking synergistic killing of PC3 cells, which have high expression levels of ALKBH3 compared to other prostate cancer cell lines (Fig 6G) [27]. Moreover, we confirmed that the ALKBH enzymes primarily mediated the combinational effect because the knockdown of ALKBH3 was sufficient to sensitize the PC3 cells to alkylating agents and abolish the effect of glutaminase inhibitor (Fig 6H). In addition, we also found that αKG plays a critical role in cell survival upon alkylating agent treatment as the addition of DM-αKG prevented MMS-induced cell death (Fig 6I). Importantly, exogenous DM-αKG abolished the ability of low glutamine to sensitize cells to MMS treatment, suggesting that glutamine deprivation sensitizes cancer cells to alkylating agent via the depletion of αKG (S6A and S6B Fig).

### Combination treatment of glutaminase inhibitor and alkylating agent suppresses tumor growth in vivo

**Fig 7:**
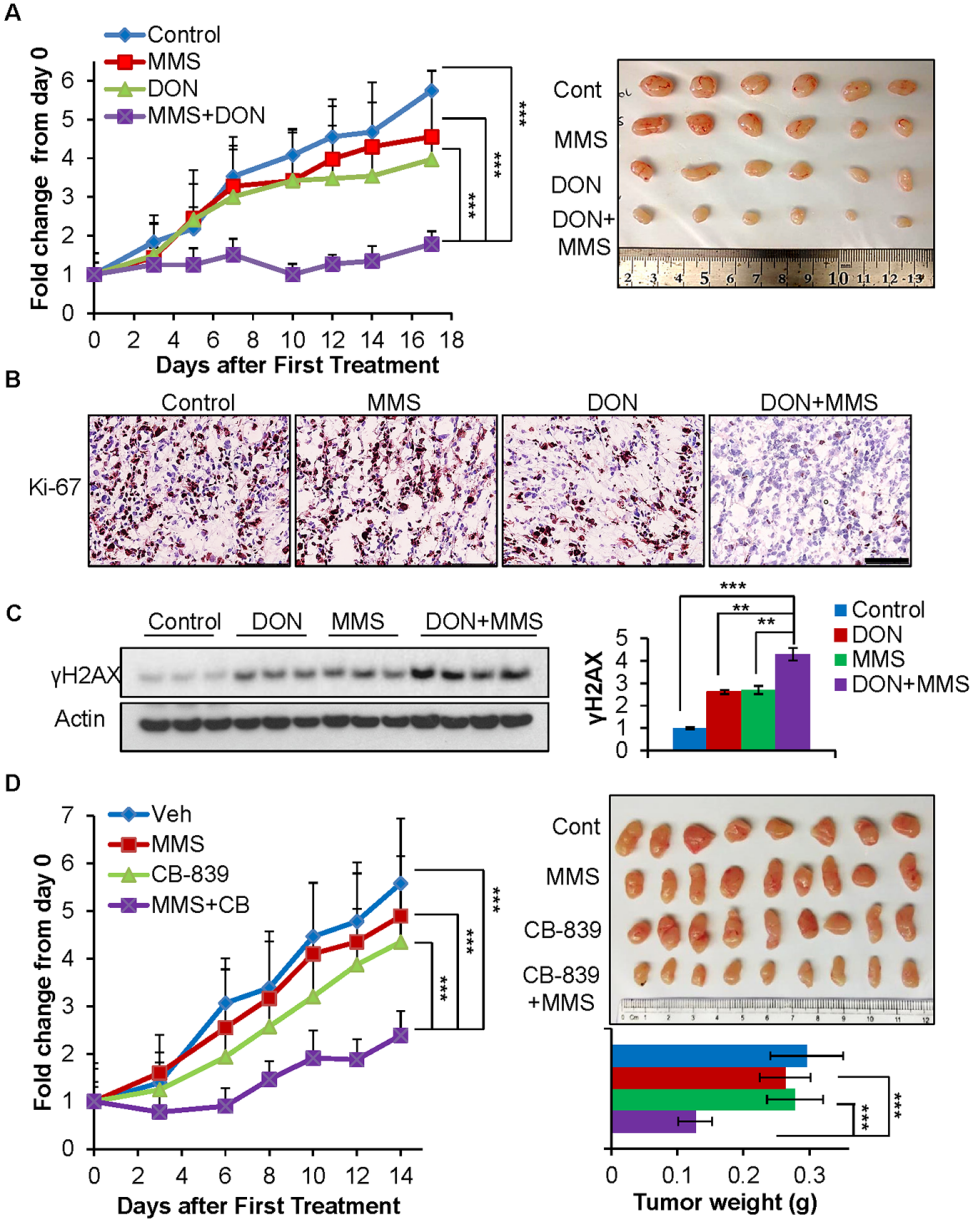
Combination treatment of glutaminase inhibitor and alkylating agent suppresses tumor growth in vivo. (A) Athymic nude mice at 7 weeks old were injected subcutaneously on the flank with 1 x 10^6^ PC3 cells. Once the tumor size reached an average of 60 mm^3^, mice were randomly placed into 4 groups and treated with PBS control (*n* = 9 tumors), 5 mg/kg DON (*n* = 8 tumors), 30 mg/kg MMS (*n* = 8 tumors), or a combination of 5 mg/kg DON and 30 mg/kg MMS (*n* = 9 tumors) 3 times per week by i.p. injection. Tumor size was measured overtime, and data represent mean ± SD, *** *P* < 0.001 by unpaired 2-tailed Student *t* test. (B) The tumors were harvested at day 18 and processed for IHC with the Ki-67 antibody. Scale bar, 100 μm. (C) The harvested tumors were lysed for immunoblotting with the indicated antibodies. Immunoblots were quantified using ImageJ and normalized to the control group; data represent mean ± SEM, ** *P* < 0.01, *** *P* < 0.001. (D) Nude mice at 6 weeks old were injected subcutaneously on the flank with 3 x 10^6^ PC3 cells. Once the tumor size reached an average of 80 mm^3^, mice were randomly placed into 4 groups and treated with vehicle control (*n* = 10 tumors), 200 mg/kg CB-839 once a day (*n* = 12 tumors), 30 mg/kg MMS 3 times per week by i.p. injection (n = 13 tumors), or a combination of CB-839 and MMS (*n* = 12 tumors). Tumor size and weight was measured overtime, and data represent mean ± SD, *** *P* < 0.001. CB, CB-839; DON, 6-Diazo-5-oxo-L-norleucine; i.p., intraperitoneal; IHC, immunohistochemistry; MMS, methyl methanesulfonate; Veh, vehicle control.

We tested whether the combinational treatment can be used to inhibit cancer growth in vivo. Athymic nude mice were used to establish xenograft tumors with alkylating agent resistant PC3 cells. Once the tumors reached an average tumor size of 60 mm^3^, the mice were treated with PBS control, DON, MMS, or a combination of DON and MMS, and the tumor volumes were measured over time. MMS treatment alone and a low dose of DON treatment did not dramatically affect tumor growth. However, the combination treatment significantly impaired tumor growth compared to the single drug treatment (Fig 7A). We also found that DON treatment significantly depleted αKG levels in tumors (S7A Fig). Additionally, IHC analysis with the proliferation marker Ki-67 revealed that combination drug treatment effectively inhibited cancer cell growth, while single drug treatment only had a marginal effect on cell proliferation, consistent with tumor growth over time (Fig 7B). While MMS or DON single treatments marginally induced γH2AX, the combination of DON and MMS treatment significantly induced γH2AX levels, suggesting that glutaminase inhibitor enhanced the DNA damage induced by the alkylating agent in vivo (Fig 7C). To further confirm the potential clinical applicability of the drug combination, we performed an additional in vivo experiment with MMS and CB-839, a specific and effective glutaminase inhibitor, which is currently in clinical trials. To determine the combinational effect on tumor growth, mice harboring tumors were treated with 200 mg/kg of CB-839 once a day, which is half the dose used in the previous publication [40]. We found that the CB-839 treatment significantly depleted αKG levels in tumors (S7B Fig). Importantly, combination treatment suppressed tumor growth and reduced tumor weight more effectively than the single drug treatment alone (Fig 7D). Together, these results suggest that inhibition of glutamine metabolism offers a substantial therapeutic benefit when combined with alkylating agents.

## Discussion

Poorly organized vasculature regions of tumors are often subjected to glutamine limitation, yet the impact of the metabolic stress on tumor development and drug response remains largely unknown. In this study, we found that glutamine deficiency inhibits ALKBH enzymes from repairing DNA alkylation damage. As a result, glutamine deficiency triggers robust DNA damage accumulation both in vitro and in vivo (Figs 1 and 2). Importantly, inhibition of glutamine metabolism with small molecule inhibitors hyper-sensitizes cancer cells to alkylating agents both in cell culture and animal models (Figs 6 and 7). Together, the study reveals previously unidentified roles of glutamine deficiency in potentiating genomic instability and modulating drug response in cancer.

We identified a novel crosstalk between glutamine metabolism and the DNA damage response. Our data reveal that glutamine deficiency, through diminishing αKG levels, inhibits the DNA repair ALKBH enzymes (Figs 3 and 4). It is well established that glutamine can be diverted to synthesize the TCA intermediate αKG in many cancer cell types to fuel the truncated TCA cycle and maintain redox homeostasis [7,41]. However, recent studies indicated that glutamine’s contribution to the TCA is both environment and tumor specific. For example, although it has been shown that glutamine is not a major source of αKG in gliomas and lung tumors [42,43], other studies support that glutamine metabolism is the main source of TCA intermediates in vivo in liver tumors, kidney tumors, and melanoma [14,44,45]. Indeed, metabolomics analysis using ^13^C-glutamine tracing have demonstrated that αKG is directly derived from glutamine in MYC-driven tumors and KRas-driven tumors [7,37]. Here, we measured αKG levels upon glutaminase inhibitor treatment in vivo and found that both DON treatment and CB-839 treatment significantly reduced intracellular αKG levels in prostate cancer xenograft tumors (S7 Fig). In addition, our results indicate that low-glutamine–mediated αKG depletion blocks the DNA repair activity of ALKBH (Figs 3 and 4). To repair DNA methylation damage, the ALKBH enzymes transfer a methyl group from the DNA adduct onto αKG and release succinate and formaldehyde as by-products [20]. We demonstrated that the reduced αKG availability is sufficient to inhibit ALKBH activity leading to both endogenous DNA alkylation damage and sensitivity to alkylating agents. Importantly, this effect is only specific to glutamine limitation, but not genotoxic stress or glucose starvation. Consistently, the oncometabolite 2-hydroxyglutarate, produced by mutated isocitrate dehydrogenase (IDH) enzymes, is an αKG structural analog that competitively binds and inhibits many αKG dependent enzymes including ALKBH [46,47].

Our results provide insights into the mechanism by which metabolic stress, such as glutamine deficiency, contributes to tumorigenesis. It is well established that glutamine metabolism via GSH production contributes to the suppression of ROS and ROS-induced DNA damage [48,49]. Surprisingly, we now show that glutamine metabolism plays an important role in maintaining genomic integrity by maintaining the ALKBH activity instead of suppressing ROS accumulation (Fig 3). A variety of alkylating agents from the environment as well as by-products of intracellular metabolism, such as S-adenosylmethionine (SAM) [39,50] often lead to intracellular alkylation damage, which is estimated to be equivalent to the exposure of 20 nM MMS [39,51]. The ALKBH enzymes contribute significantly to the repair of these sites of alkylation damage, as ALKBH3 knockdown alone is sufficient to induce alkylated DNA adducts in some cancers, as demonstrated in previous reports and Fig 4 [27]. Our data indicate that glutamine deficiency in tumors inhibits the repair of endogenous DNA damage mediated by the ALKBH enzymes leading to the accumulation of alkylated DNA adducts and DSBs. Thus, metabolic stress, such as glutamine deficiency, may contribute to genomic instability and promote tumorigenesis.

Lastly, our study provides an important molecular basis for combinational therapy to sensitize cells to alkylating agent treatment using glutaminase inhibitors. Alkylating agents remain important frontline antineoplastic drugs for many highly aggressive and metastatic cancers despite high toxicity and carcinogenic potential [39]. However, malignant cells often become resistant to such treatments due to robust DNA repair machineries [52,53]. For example, ALKBH enzymes have been shown to be overexpressed in some human cancers to promote drug resistance and tumor progression [24]. Our study demonstrates that targeting glutamine metabolism inhibits the DNA repair activity of the ALKBH enzymes and sensitizes cancer cells to alkylating agent treatment. Therefore, this novel combination drug treatment can improve efficacy and minimize the side effects of alkylating agents. In addition, glutaminase inhibitor treatment, by indirectly inhibiting the ALKBH enzymes, may serve as an effective strategy to block the oncogenic function of ALKBH enzymes and restore cancer cell sensitivity to alkylating agents. Moreover, a new glutaminase inhibitor, CB-839, is well tolerated in patients and has passed phase 1 clinical trials for multiple cancers [54,55]. Our data here provide a new direction to use CB-839 clinically to sensitize cancer cells, particularly cancers with ALKBH overexpression, to alkylating agents.

## Materials and methods

### Ethics statement

All animal work has been conducted according to the approved Institutional Animal Care and Use Committee (IACUC) protocols at the City of Hope Cancer Center (11011).

### Cell culture and reagents

3T3-MEF and HT1080 cells were purchased from American Type Culture Collection (ATCC). HCT116 p53^-/-^, Ras transformed 3T3-MEF cells, and M229 have been generated previously [14, 56]. *Bak*^-/-^ /*Bax*^-/-^ MEF and paired wild-type MEF were gifts from Dr. Craig Thompson laboratory at Sloan-Kettering Cancer Center [30]. *Alkbh3*^-/-^ MEF and paired wild-type MEF were obtained from Dr. Timothy O’Connor laboratory at City of Hope [57]. These cells were cultured in Dulbecco's Modified Eagle Medium (DMEM, Corning) supplemented with 10% fetal bovine serum (FBS; Gemini Bio-Products, West Sacramento, CA), 100 units/mL of penicillin, and 100 μg/mL of streptomycin (Gemini Bio-Products). PC3, EB3, Namalwa, DB, and CA46 were purchased from ATCC and cultured in RPMI 1640 medium supplemented with 10% FBS and penicillin/streptomycin. All cells were cultured at 37°C with 5% CO_2_. Cells were routinely tested for mycoplasma contamination using MycoAlert Mycoplasma Detection Kit.

The reagents L-DON, BPTES, CPT, Doxo, NAC, MMS, and DM-αKG were purchased from Sigma-Aldrich (St. Louis, MO). GSH monoethyl ester (353905) was purchased from Calbiochem (Sigma-Aldrich). CCNU and CB-839 were purchased from Selleckchem (Houston, TX).

### Glutamine and amino acid starvation

Glutamine starvation was described previously [16]. Briefly, cells were washed once with PBS and cultured in complete medium or glutamine-free medium. For the glucose starvation experiment, DMEM without glucose (11966; Invitrogen, Carlsbad, CA) was supplemented with 10% dialyzed FBS (Gemini) to make glucose-free medium. Glucose (Sigma) was added back to the glucose-free medium to make the complete medium (25 mM) or low-glucose medium (1 mM). For the amino acid starvation experiments, DMEM Ham’s F-12 medium without amino acids (D9811-01; US Biological, Salem, MA) was supplemented with 10% dialyzed FBS. Different amino acids (purchased from Sigma) were added back to amino acid–free medium to make a medium without glutamine, serine, asparagine, aspartate, arginine, leucine, or glycine. To make medium without essential amino acid, the amino acid-free medium was supplemented with nonessential amino acid solution (Sigma M7145) and 4 mM L-glutamine.

### Flow cytometry

To assess cell death, cells after treatment were washed twice with PBS and stained with AnnexinV and/or PI (eBioscience, Thermo Fisher Scientific, Waltham, MA) for 10 minutes at room temperature and processed according to the manufacturer’s protocol. For ROS measurement, cells after treatment were washed once with PBS and incubated with 2.5 μM Dihydroethidium (Thermo Fisher Scientific) and DAPI for 20 minutes at 37°C. Flow analysis was carried out using the 9-color CyAn ADP from Beckman Coulter (Miami, FL)

### Cell proliferation and viability assay

Cell viability was determined by MTS assay using CellTiter 96 AQueous MTS Reagent Powder (Promega, Durham, NC). 2mg/ml 3-(4,5-dimethylthiazol-2-yl)-5-(3-carboxymethoxyphenyl)-2-(4-sulfophenyl)-2H-tetrazolium (MTS) and 0.92 mg/ml phenazine methosulfate (PMS) were mixed in the ratio 20:1 to make the 10 x stock solution. The mixture was added directly to the cells in a 96-well plate followed by incubation at 37°C for 1 to 4 hours. The absorbance was measured at 490 nm. Cell proliferation was determined by CellTiter Glo assay (Promega) according to the manufacturer’s protocol.

### Western blotting

Western blotting was carried out as previously described [15]. Briefly, cells after treatment were washed three times with PBS and lysed in precooled RIPA buffer supplemented with protease inhibitor (Roche) and phosphatase inhibitor (Thermo Fischer Scientific). The cells lysates were then sonicated briefly, and protein concentration was determined by BCA protein assay kit (Thermo Fischer Scientific). The protein lysates were loaded on precast NuPAGE Bis-Tris Gels (Life Technologies, Durham, NC) followed by transfer onto nitrocellulose. Immunoblotting was performed with the following antibodies: γH2AX (Cell Signaling Technology [CST], 9718 and 2577; Danvers, MA), Actin (Sigma-Aldrich), Cleaved Caspase 3 (CST, 9665), Cleaved PARP (CST, 5625), ALKBH3 (Santa Cruz Biotechnology, Dallas, TX), Bak (CST, 5023), p-ATM (CST, 5883), p-BRCA (CST, 9009), p-Chk1 (CST, 2348), and GAPDH (CST, 5174).

### siRNA knockdown

Smartpool On-target plus human ALKBH3 siRNA from Dharmacon (L-004289-00-0005; Lafayette, CO) was used to transiently knock down ALKBH3 protein in PC3 cells. Cells were transfected with ALKBH3 siRNA or control siRNA (Dharmacon) using RNAi Max lipofectamine reagent (Invitrogen) according to the manufacturer’s protocol. Two days after the first transfection, the cells were transfected again with the siRNA to increase transfection efficiency. Glutamine deprivation or drug treatment was performed 2 days post the second transfection.

### Dot blot assay

Dot blot assay was performed as described previously [27]. Briefly, genomic DNA was isolated after treatment using DNeasy Blood & Tissue Kit (Qiagen, Hilden, Germany). DNA concentrations were measured by Nanodrop and equal amounts of DNA were loaded onto positively charged nylon membrane (Amersham Hybond-N+; GE Healthcare, Little Chalfont, United Kingdom). After UV cross-linking and blocking with 5% milk, the membranes were incubated with anti-3meC antibody (1:2,000 dilution, Active Motif, 61179) at 4°C with shaking overnight. The membrane was then probed with horseradish-peroxidase-conjugated anti-rabbit secondary antibodies. The dot blot signal was visualized using Western Lightning Plus-ECL (PerkinElmer, Waltham, MA). For the methylene blue staining, the membrane was incubated with 0.02% methylene blue in 0.3 M sodium acetate (pH 5.2) with shaking for 10 minutes at room temperature.

### Immunofluorescence and IHC

Immunofluorescence was performed as described previously [58]. Briefly, cells were fixed with 4% formaldehyde, blocked with 1% BSA followed by staining with antibodies against γH2AX (NB100-78356; Novus Biologicals, Littleton, CO), 53BP1 (ab3682; Abcam, Cambridge, United Kingdom), cleaved caspase-3 (CST, 9661), and DAPI. Secondary antibodies, goat anti-rabbit Alexa Fluor 488 and goat anti-mouse Alexa Fluor 594, were purchased from MilliporeSigma (St. Louis, MO). Images were captured 20x or 40x magnification by using a Zeiss LSM 700 Confocal Microscope and by using the ZEN Blue image acquisition software. IHC assays were performed as previously described [14]. The staining was performed with the following antibodies: γH2AX (CST, 9718) and Ki67 (Dako; Fisher Scientific). Images were acquired by using Olympus BX50 Upright Microscope at 20x magnification.

### Metabolite extraction and mass spectrometry

Mass spectrometry was performed as previously described [14]. After amino acid starvation, medium was aspirated quickly and precooled 80% methanol was added directly to the cells. The cells were then incubated at −80°C for 15 minutes followed by centrifugation at 4°C. The supernatant was then dried with a speed vacuum and the extracted metabolite pellet was stored at −80°C. Liquid chromatography–mass spectrometry (LC-MS) and data analysis were processed at Duke University (Durham, NC) and described previously [59]. Intracellular αKG levels were also measured using Alpha Ketoglutarate Assay Kit (Abcam) according to the manufacturer’s protocol for colorimetric assay without the deproteinization step.

### ALKBH enzymatic activity assay

DNA repair reactions were performed with the recombinant ALKBH3 proteins and oligonucleotide substrates in the reaction buffer (50 mM Hepes-KOH, 40 mM αketoglutarate, 2 mM Ascorbate, 40 mM FeSO4) for 1 hour at 37°C. The 3meC containing oligonucleotide substrate is 5′AAAGCAAGCAGATYATTCGAAAAAGCGAAA-3′, Y = 3-meC. The complementary oligonucleotide with fluorescent tag is 5′TTTCGCTTTTTCGAATGATCTGCTTGCTTT-Cy5.5–3′. The oligonucleotide substrate and the complementary oligonucleotide were mixed and heated to 90°C for 1 minute and cooled at 1°C /minute to 4°C to anneal. The reactions were then digested with DpnII restriction enzyme and the products were separated by 20% nondenaturing TBE-PAGE gel.

### Mouse xenografts

For glutaminase inhibitor treatment, 7-week-old athymic nude male mice (Taconic Laboratories, Rensselaer, NY) were injected subcutaneously on the flank with 1 x 10^6^ cells of PC3, HT1080, or HCT116 p53^-/-^. Once the tumors were established, the mice were treated with PBS control or 5–15 mg/kg DON as indicated 3 times per week. For combination drug treatment of MMS and DON, 7-week-old athymic nude male mice were injected subcutaneously on the flank with 1 x 10^6^ PC3 cells. After the tumors were established, mice were randomly placed into 4 groups and treated with PBS control, 5 mg/kg DON, 30 mg/kg MMS, or a combination of 5 mg/kg DON and 30 mg/kg MMS 3 times per week. For combination drug treatment of MMS and CB-839, 6-week-old nude male mice were injected subcutaneously on the flank with 3 x 10^6^ PC3 cells. After the tumors were established, mice were randomly placed into 4 groups and treated with vehicle control, 200 mg/kg CB-839 prepared in vehicle by oral gavage once a day, 30 mg/kg MMS by intraperitoneal injection 3 times per week, or a combination of CB-839 and MMS. The vehicle consisted of 5% DMSO in corn oil (Sigma-Aldrich). The tumor size was measured every other day and tumor volume was calculated by using the formula 0.5 (width2 × length). All studies involving animals were performed according to approved IACUC protocols at the City of Hope Cancer Center.

### Statistical analysis

Results are shown as averages; error bars represent either the SEM or SD as indicated. The unpaired Student *t* test was used to determine the statistical significance of differences between means (* *P* < 0.05, ** *P* < 0.01, *** *P* < 0.001).

## Supporting information

S1 Fig Glutamine deficiency induces DNA damage independent of cell death. (A) EB3 cells were cultured in cell medium with the indicated glutamine concentration for 48 hours. (B) EB3 cells were cultured in glutamine-free medium or complete medium for the indicated time up to 48 hours; cells were lysed for immunoblotting (C) MEF cells were cultured in complete or glutamine-free medium for 48 hours. Cells were fixed for immunofluorescence using the indicated antibodies. Scale bar 100μm. (D) Bak-/- /Bax -/- MEF and littermate wild-type MEF cells were treated with 3.4 μM doxorubicin overnight; cells were lysed for western blot analysis using the indicated antibodies. MEF, mouse embryonic fibroblast.
(TIF)

S2 Fig Glutaminase inhibitor treatment depletes intracellular αKG levels in vitro. MEF cells were treated with 50 μM BPTES, 25 μM CB-839, or 50 μM DON for 48 hours. Relative intracellular αKG levels were determined using an αKG assay kit, and normalized to the protein level. Data represent mean ± SD of 3 independent cell cultures (*** *P* < 0.001). αKG, alpha-ketoglutarate; DON, 6-Diazo-5-oxo-L-norleucine; MEF, mouse embryonic fibroblast.
(TIF)

S3 Fig Glutamine deficiency inhibits the ALKBH enzymes leading to DNA damage accumulation. (A) PC3 cells were transfected with ALKBH3 siRNA or control siRNA. Two days after transfection, control PC3 cells and ALKBH3 knockdown cells were cultured in complete or glutamine-free medium for 3 days; genomic DNA was extracted to perform dot blot analysis using the 3meC specific antibody. (B) Wild-type MEF, Alkbh2-/- MEF or Alkbh3-/- MEF cells were cultured in complete or glutamine-free medium overnight. Cells were lysed for immunoblotting using the indicated antibodies. (C) PC3 cells were transfected with ALKBH siRNA or control siRNA twice. Four days after siRNA transfection, control cells and ALKBH3 knockdown cells were treated with 0.1 μM CPT overnight; cells were fixed for immunofluorescence using the indicated antibodies. Scale bar 20 μm. Data represent mean ± SD from 2 independent cell cultures, ** *P* < 0.01; shown is the percentage of cells showing >10 foci. ALKBH, alkylation repair homolog; ALKBH3, AlkB homolog 3; CPT, camptothecin; MEF, mouse embryonic fibroblast; siRNA, small interfering RNA.
(TIF)

S4 Fig Exogenous αKG fails to rescue low glutamine-induced DNA damage in Alkbh deficient cells. (A) Wild-type MEF, Alkbh2-/- MEF or Alkbh3-/- MEF cells were cultured in completed, glutamine-free medium or glutamine-free medium supplemented with 3.5 mM αKG for 12 hours. Cells were lysed for immunoblotting using the indicated antibodies. (B) PC3 cells were transfected with ALKBH3 siRNA twice. Four days after transfection, control PC3 cells and ALKBH3 knockdown cells were cultured in complete, glutamine-free medium or glutamine-free medium supplemented with 3.5 mM DM-αKG for 2 days; cells were lysed for immunoblotting using the indicated antibodies. αKG, alpha-ketoglutarate; ALKBH3, AlkB homolog 3; DM-αKG, dimethyl-αKG; MEF, mouse embryonic fibroblast; siRNA, small interfering RNA.
(TIF)

S5 Fig Inhibition of glutamine metabolism does not sensitize cell to other classes of chemotherapy drug. (A) Ras-transformed MEF cells were treated with the indicated concentration of Doxo alone or in combination with 20 μM BPTES for 48 hours. (B) Ras-transformed MEF cells were treated with the indicated concentration of CPT alone or in combination with 20 μM BPTES for 48 hours. Relative cell survival was assessed by MTS assay and normalized to the control. Data represent mean ± SD of 3 independent cell cultures. CPT, camptothecin; Doxo, doxorubicin; MEF, mouse embryonic fibroblast.
(TIF)

S6 Fig Glutamine deprivation sensitizes cells to alkylating agent through the depletion of αKG. (A) MEF cells were cultured in complete (control) media, glutamine-free medium or glutamine-free medium supplemented with 3.5 mM DM-αKG overnight. Intracellular αKG levels were measured by an αKG assay kit and normalized to total protein levels. Data represent mean ± SD of 3 independent cell cultures. (** *P* < 0.01,*** *P* < 0.001). (B) MEF cells were treated with 2 mM MMS for 1 hour, washed, and subsequently cultured in complete medium, complete medium supplemented with 3.5 mM DM-αKG, low (0.1 mM) glutamine medium, or low glutamine medium supplemented with 3.5 mM DM-αKG for 12 hours. Relative survival was determined by MTS assay normalized to the control of each group. Data represent mean ± SD of 3 independent cell cultures (** *P*< 0.01). αKG, alpha-ketoglutarate; DM-αKG, dimethyl-αKG; MEF, mouse embryonic fibroblast; MMS, methyl methanesulfonate.
(TIF)

S7 Fig Glutaminase inhibitor treatment depletes intracellular αKG levels in vivo. Control tumors, DON-treated tumors (A), or CB839 treated tumors (B) from Fig 7A and 7D were lysed, and αKG levels relative to the control were determined using an αKG assay kit and normalized to the tumor weight. Data represent mean ± SEM of 4 different tumors. (* *P* < 0.05, ** *P* < 0.01, *** *P* < 0.001). αKG, alpha-ketoglutarate; DON, 6-Diazo-5-oxo-L-norleucine.
(TIF)

S1 Data Additional data used in the generation of the figures in the manuscript and supporting information. (XLSX)

## Abbreviations

3meC: 3-methyl cytosine
αKG: alpha-ketoglutarate
ALKBH: AlkB homolog
ATCC: American Type Culture Collection
BPTES: Bis-2-(5-phenylacetamido-1,3,4-thiadiazol-2-yl)ethyl sulfide
CCNU: lomustine
CPT: camptothecin
CST: Cell Signaling Technology
DM-αKG: dimethyl-αKG
DON: 6-Diazo-5-oxo-L-norleucine
Doxo: doxorubicin
DSB: double strand breaks
FBS: fetal bovine serum
GSH: glutathione
IACUC: Institutional Animal Care and Use Committee
IDH: isocitrate dehydrogenase
IHC: immunohistochemistry
LC-MS: liquid chromatography–mass spectrometry
MEF: mouse embryonic fibroblast
MMS: methyl methanesulfonate
NAC: N-acetyl cysteine
PARP: poly (adenosine diphosphate-ribose) polymerase
PMS: phenazine methosulfate
ROS: reactive oxygen species
SAM: S-adenosylmethionine
TCA: tricarboxylic acid
