# Supplementary figures and images for: Glutamine deficiency induces DNA alkylation damage and sensitizes cancer cells to alkylating agents through inhibition of ALKBH enzymes
Source: PLoS Biol. 2017 Nov 6;15(11):e2002810. doi: 10.1371/journal.pbio.2002810 (PMC5673162; doi:10.1371/journal.pbio.2002810)

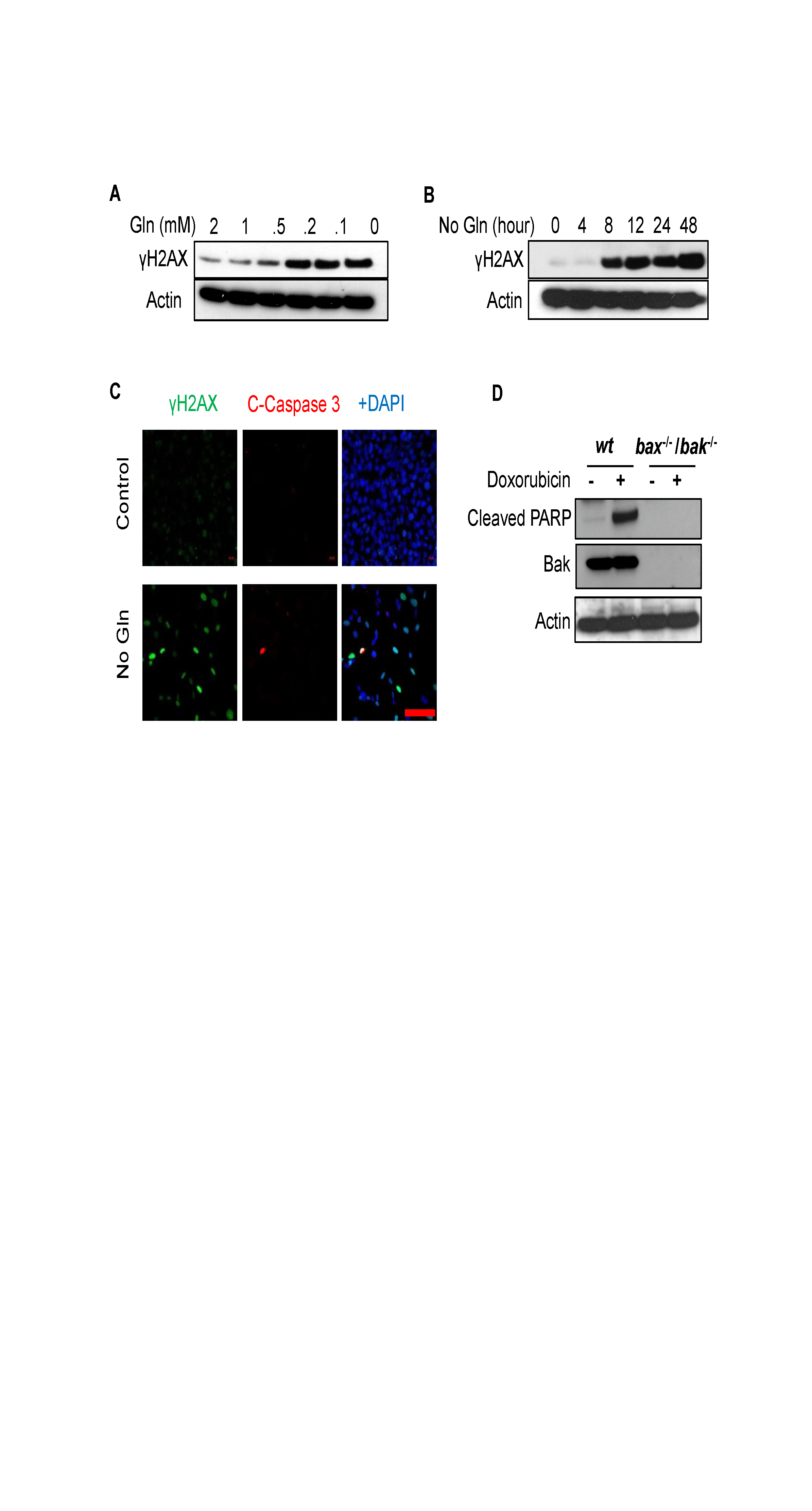

Supplement: S1 Fig — (A) EB3 cells were cultured in cell medium with the indicated glutamine concentration for 48 hours. (B) EB3 cells were cultured in glutamine-free medium or complete medium for the indicated time up to 48 hours; cells were lysed for immunoblotting (C) MEF cells were cultured in complete or glutamine-free medium for 48 hours. Cells were fixed for immunofluorescence using the indicated antibodies. Scale bar 100μm. (D) Bak-/- /Bax -/- MEF and littermate wild-type MEF cells were treated with 3.4 μM doxorubicin overnight; cells were lysed for western blot analysis using the indicated antibodies. MEF, mouse embryonic fibroblast. (TIF) [file pbio.2002810.s001.tif]

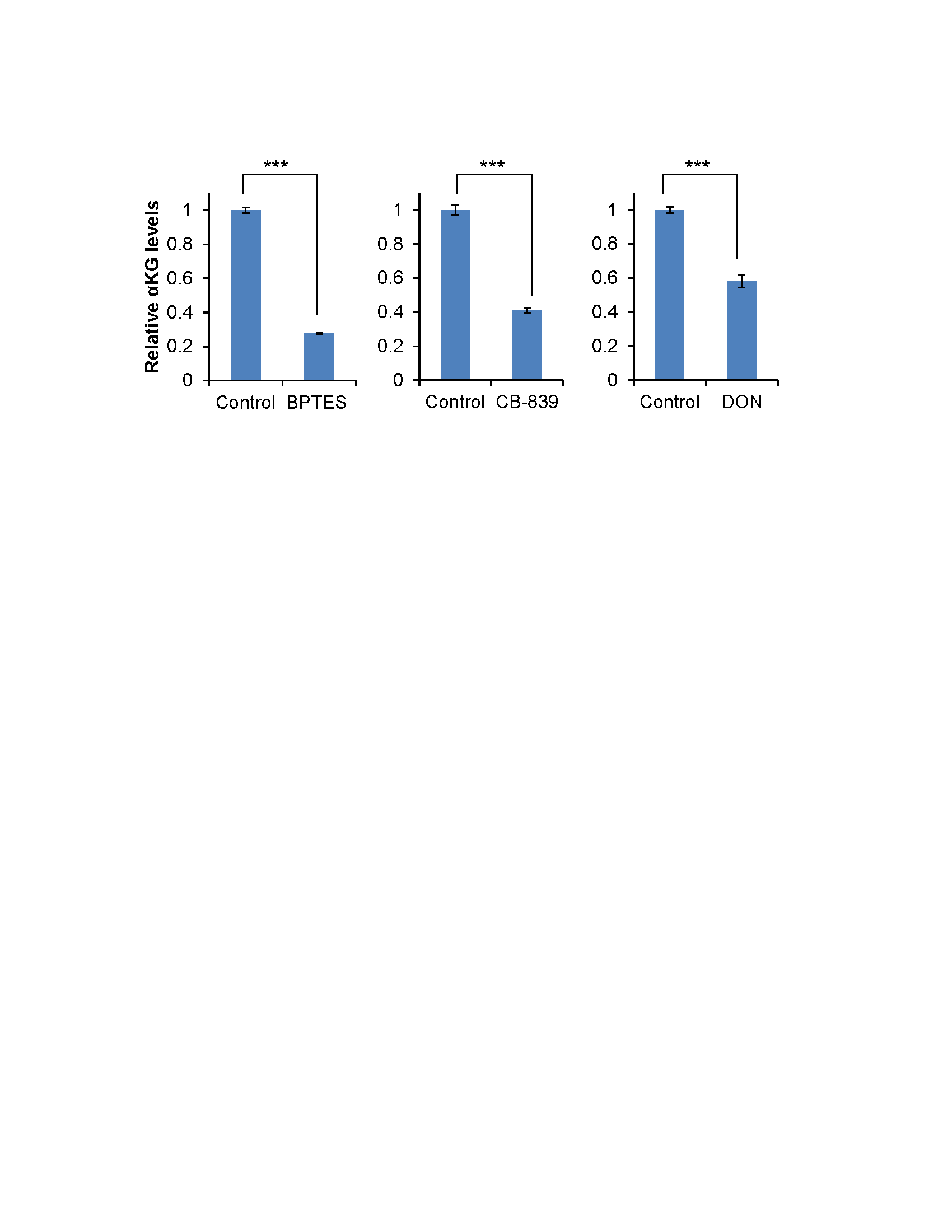

Supplement: S2 Fig — MEF cells were treated with 50 μM BPTES, 25 μM CB-839, or 50 μM DON for 48 hours. Relative intracellular αKG levels were determined using an αKG assay kit, and normalized to the protein level. Data represent mean ± SD of 3 independent cell cultures (*** P < 0.001). αKG, alpha-ketoglutarate; DON, 6-Diazo-5-oxo-L-norleucine; MEF, mouse embryonic fibroblast. (TIF) [file pbio.2002810.s002.tif]

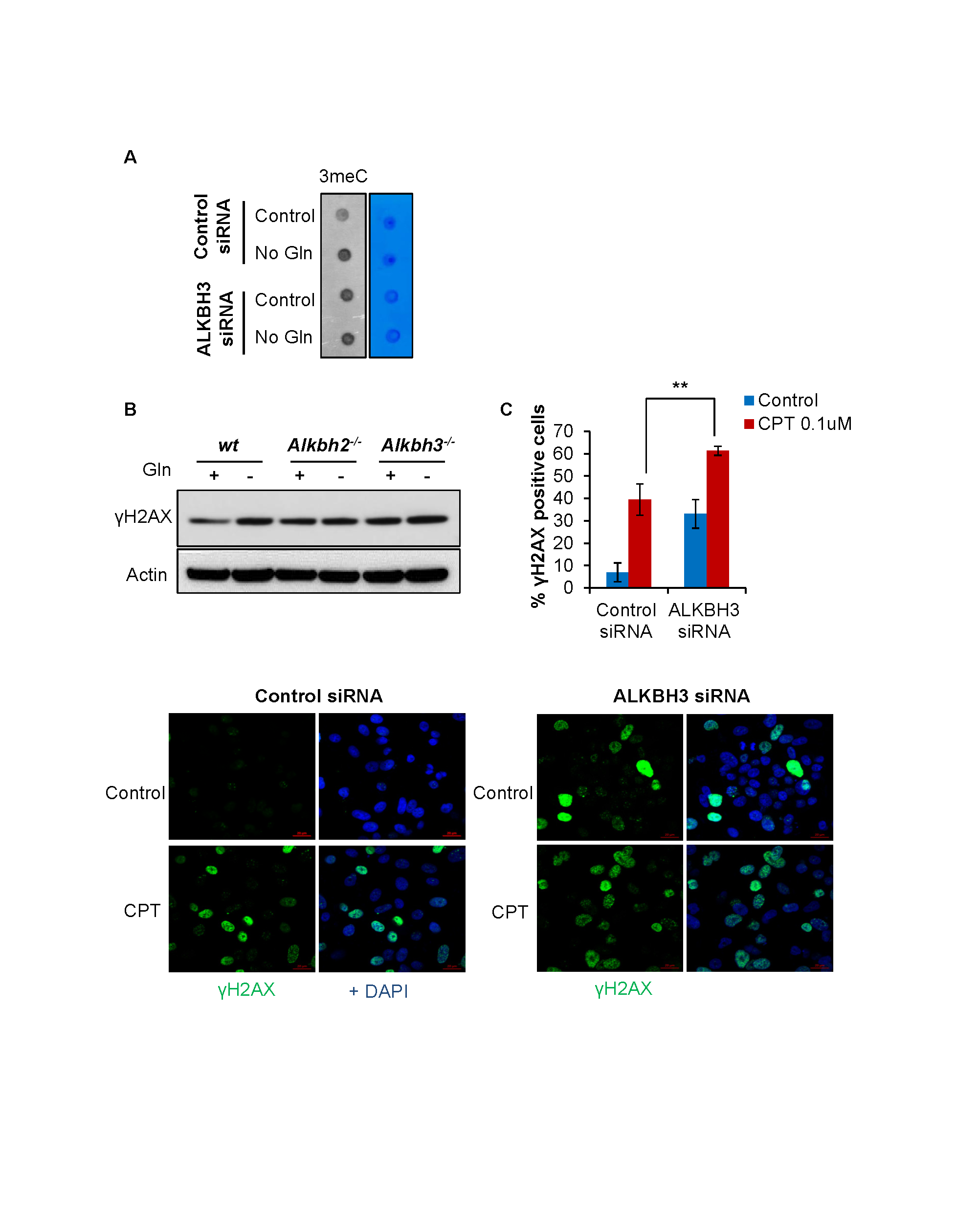

Supplement: S3 Fig — (A) PC3 cells were transfected with ALKBH3 siRNA or control siRNA. Two days after transfection, control PC3 cells and ALKBH3 knockdown cells were cultured in complete or glutamine-free medium for 3 days; genomic DNA was extracted to perform dot blot analysis using the 3meC specific antibody. (B) Wild-type MEF, Alkbh2-/- MEF or Alkbh3-/- MEF cells were cultured in complete or glutamine-free medium overnight. Cells were lysed for immunoblotting using the indicated antibodies. (C) PC3 cells were transfected with ALKBH siRNA or control siRNA twice. Four days after siRNA transfection, control cells and ALKBH3 knockdown cells were treated with 0.1 μM CPT overnight; cells were fixed for immunofluorescence using the indicated antibodies. Scale bar 20 μm. Data represent mean ± SD from 2 independent cell cultures, ** P < 0.01; shown is the percentage of cells showing >10 foci. ALKBH, alkylation repair homolog; ALKBH3, AlkB homolog 3; CPT, camptothecin; MEF, mouse embryonic fibroblast; siRNA, small interfering RNA. (TIF) [file pbio.2002810.s003.tif]

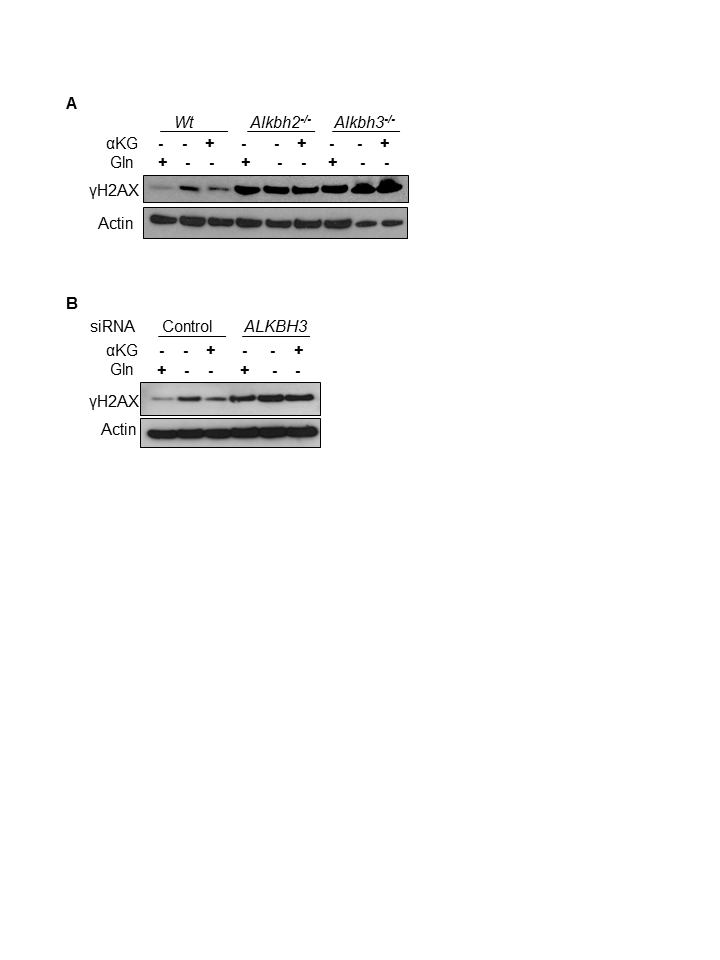

Supplement: S4 Fig — (A) Wild-type MEF, Alkbh2-/- MEF or Alkbh3-/- MEF cells were cultured in completed, glutamine-free medium or glutamine-free medium supplemented with 3.5 mM αKG for 12 hours. Cells were lysed for immunoblotting using the indicated antibodies. (B) PC3 cells were transfected with ALKBH3 siRNA twice. Four days after transfection, control PC3 cells and ALKBH3 knockdown cells were cultured in complete, glutamine-free medium or glutamine-free medium supplemented with 3.5 mM DM-αKG for 2 days; cells were lysed for immunoblotting using the indicated antibodies. αKG, alpha-ketoglutarate; ALKBH3, AlkB homolog 3; DM-αKG, dimethyl-αKG; MEF, mouse embryonic fibroblast; siRNA, small interfering RNA. (TIF) [file pbio.2002810.s004.tif]

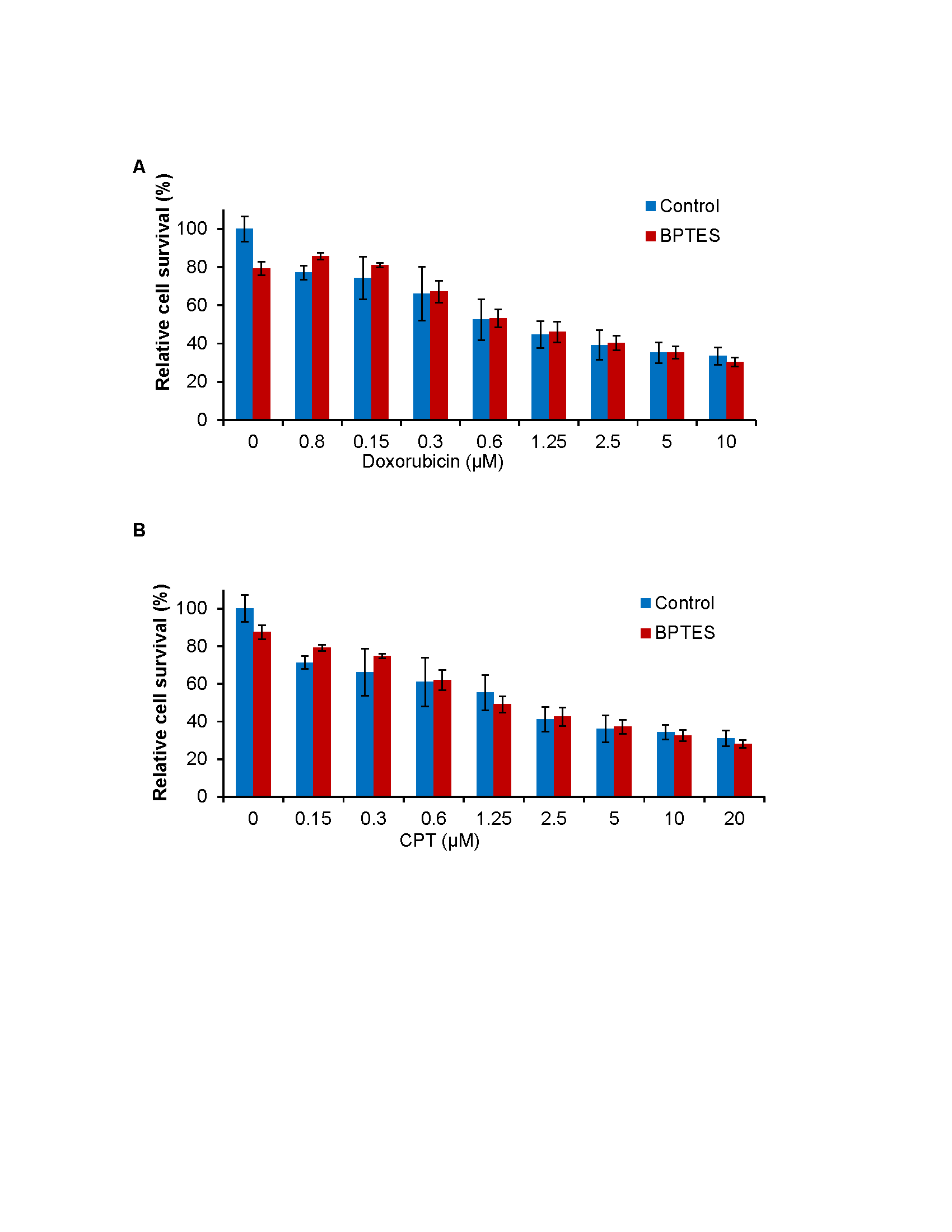

Supplement: S5 Fig — (A) Ras-transformed MEF cells were treated with the indicated concentration of Doxo alone or in combination with 20 μM BPTES for 48 hours. (B) Ras-transformed MEF cells were treated with the indicated concentration of CPT alone or in combination with 20 μM BPTES for 48 hours. Relative cell survival was assessed by MTS assay and normalized to the control. Data represent mean ± SD of 3 independent cell cultures. CPT, camptothecin; Doxo, doxorubicin; MEF, mouse embryonic fibroblast. (TIF) [file pbio.2002810.s005.tif]

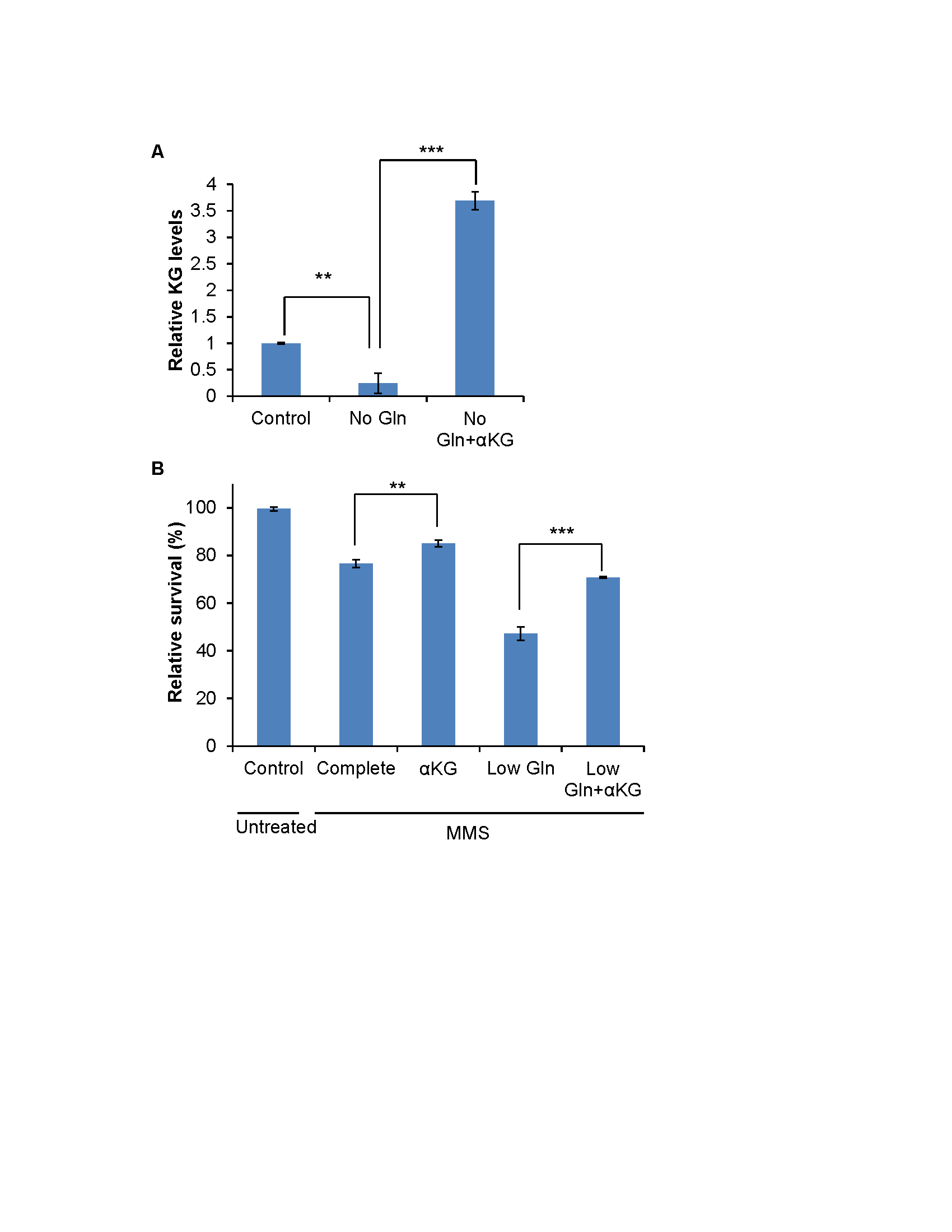

Supplement: S6 Fig — (A) MEF cells were cultured in complete (control) media, glutamine-free medium or glutamine-free medium supplemented with 3.5 mM DM-αKG overnight. Intracellular αKG levels were measured by an αKG assay kit and normalized to total protein levels. Data represent mean ± SD of 3 independent cell cultures. (** P < 0.01,*** P < 0.001). (B) MEF cells were treated with 2 mM MMS for 1 hour, washed, and subsequently cultured in complete medium, complete medium supplemented with 3.5 mM DM-αKG, low (0.1 mM) glutamine medium, or low glutamine medium supplemented with 3.5 mM DM-αKG for 12 hours. Relative survival was determined by MTS assay normalized to the control of each group. Data represent mean ± SD of 3 independent cell cultures (** P< 0.01). αKG, alpha-ketoglutarate; DM-αKG, dimethyl-αKG; MEF, mouse embryonic fibroblast; MMS, methyl methanesulfonate. (TIF) [file pbio.2002810.s006.tif]

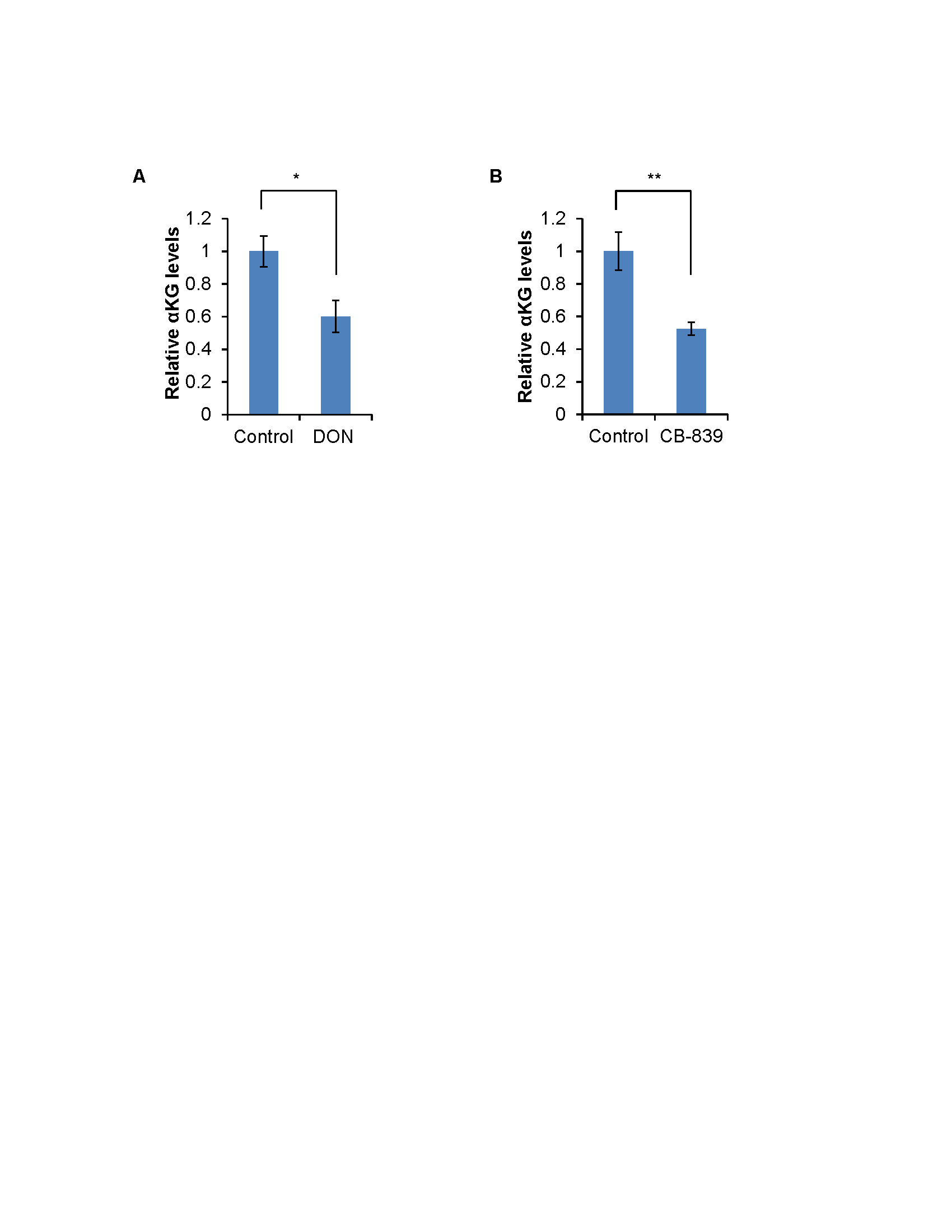

Supplement: S7 Fig — Control tumors, DON-treated tumors (A), or CB839 treated tumors (B) from Fig 7A and 7D were lysed, and αKG levels relative to the control were determined using an αKG assay kit and normalized to the tumor weight. Data represent mean ± SEM of 4 different tumors. (* P < 0.05, ** P < 0.01, *** P < 0.001). αKG, alpha-ketoglutarate; DON, 6-Diazo-5-oxo-L-norleucine. (TIF) [file pbio.2002810.s007.tif]
